# Exposure to violence is associated with decreased neural connectivity in emotion regulation and cognitive control, but not working memory, networks after accounting for socioeconomic status: a preliminary study

**DOI:** 10.1186/s12993-022-00201-8

**Published:** 2022-12-12

**Authors:** Samantha R. Mattheiss, Hillary Levinson, Miriam Rosenberg-Lee, William W. Graves

**Affiliations:** 1 Psychology Department, Felician University, Lodi, NJ USA; 2 Psychology Department, Rutgers University - Newark, Newark, NJ USA

**Keywords:** Violence, Connectivity, Resting state, ACC, dlPFC, Amygdala, Emotion regulation, Working memory, Adversity

## Abstract

Previous research has demonstrated behavioral and neural differences associated with experiencing adversity. However, adversity is unlikely to be a monolithic construct, and we expect that examining effects of more specific components such as exposure to violence in the home community will yield more concretely interpretable results. Here we account for effects of low socioeconomic status (SES) to examine the specific effects of exposure to violence on functional connectivity between brain areas known to be related to emotion regulation and working memory. Decreased resting state functional connectivity for individuals exposed to high compared to low levels of violence during childhood was predicted for two sets of areas: (1) bilateral amygdala with anterior medial regions involved in cognitive control of emotion, and (2) the right dorsolateral prefrontal cortex (dlPFC) with frontal and parietal regions implicated in working memory. Consistent with our predictions, increasing exposure to violence was related to decreased resting state functional connectivity between the right amygdala and anterior cingulate cortex, even after accounting for SES. Also after accounting for SES, exposure to violence was related to reductions in connectivity between the right dlPFC and frontal regions, but not with parietal regions typically associated with working memory. Overall, this pattern suggests increased exposure to violence in childhood is associated with reduced connectivity among key areas of the circuitry involved in emotion regulation and cognitive control, but not working memory. These results offer insight into the neural underpinnings of behavioral outcomes associated with exposure to violence, laying the foundation for ultimately designing interventions to address the effects of such exposure.

**Supplementary Information:**

The online version contains supplementary material available at 10.1186/s12993-022-00201-8.

## Background

Both witnessing and being a victim of violence have been linked to a wide range of adverse effects including detrimental effects on mental health [1], poorer performance on academic assessments [2], cognitive deficits [3] and emotion and behavioral dysregulation [4, 5]. Some of the neuroimaging work on exposure to violence has utilized experimental manipulations of acute exposure to violence during, for example, violent video games, video clips displaying media violence, and images with violent content [6–8]. Other studies have used various measures of exposure to violence, such as self-reported violence in the home or experiences of childhood maltreatment, to predict task-based [9, 10] and resting-state [11] connectivity in children and adolescents. No studies, to our knowledge, have explored the cumulative effects of childhood exposure to community violence on adult resting state connectivity of circuits underlying emotion regulation and working memory. Resting state connectivity has been shown to be a measure of overall brain organization and communication between regions [12–14]. Thus, our study contributes to the literature on the neural circuitry of adversity by examining the long-term effects of exposure to violence on well-characterized brain circuits involved in emotion regulation [15, 16].

Exposure to violence often co-occurs with low socioeconomic status (SES), as well as several other SES-related factors including maternal deprivation, poor access to health care, and exposure to noise and toxins [17]. Research has broadly demonstrated adverse effects of such factors on affective and cognitive functioning, including impairments in working memory and emotion regulation, [17–21]. Despite it being a challenging undertaking, distinguishing the effects of factors that commonly coincide with low SES is essential to determining how to prioritize intervention efforts. For example, interventions focused on alleviating poverty may not address systemic factors, like schooling disparities and exposure to violence. The use of neuroimaging methods has the potential to elucidate the neural consequences of specific factors, such as exposure to violence, thus providing even more precise information on which to ultimately base the development of novel interventions. The current study aims to identify precise differences in resting state connectivity in relation to exposure to community violence, above and beyond the effects of SES.

### Exposure to violence: effects on emotion regulation circuitry

Adversity, a construct that includes, but is not limited to, exposure to violence, has been shown to have detrimental effects on emotion regulation [20, 22–24]. Task-based neuroimaging studies demonstrate the effects of childhood adversity on amygdala reactivity to threatening stimuli [20, 25–27]. Increased responsivity to threat is thought to be related to decreased regulatory activity from the prefrontal cortex (PFC) [25], shown to regulate threat and reward responses by influencing the amygdala. Specifically, increased amygdala activation corresponds to decreased PFC activity [28–32]. Adversity has been linked not only to emotion regulation deficits and increased amygdala reactivity, but also to decreased fronto-amygdala connectivity [11, 23, 33–35], yet to date, few neuroimaging studies have aimed to disaggregate dimensions of adversity [23]. Neurobiological influences, such as increased release of catecholamines, including norepinephrine, are thought to underly such changes in connectivity [36].

Several studies have shown similar effects from exposure to violence—a subtype of adversity [5, 37]. Behaviorally, children who have been exposed to violence have a decreased ability to regulate negative emotional responses, resulting in impairments in goal-directed behavior in the presence of threatening stimuli [3, 38–40]. Neuroscientific research is consistent with these behavioral findings, with childhood maltreatment and violence exposure in the family shown to be related to increased neural amygdala response to threat [41, 42]. Also in line with adversity-induced reduction in prefrontal-amygdala connectivity, children who were exposed to physical, sexual, or domestic violence had reduced activation of the anterior cingulate cortex (ACC) while viewing fearful faces [43], relative to peers who had not been exposed to violence. The ACC is involved in a diverse range of cognitive and affective functions, including, but not limited to, emotion regulation (e.g. [44, 45], conflict monitoring and error detection [46–48], and avoidance of threat cues [43]. Moreover, the prefrontal cortex has been shown to be impacted by adversity. For example, an acute manipulation of violence exposure—playing violent video games—also corresponded to reduced prefrontal cortex activity during inhibition tasks [49]. Finally, in a study most comparable to ours, urban youth ages 9–15 who reported having experienced at least one form of trauma compared to those who reported not having experienced trauma had less amygdala-prefrontal functional connectivity [11]. However, in that study, the trauma-exposed group reported lower levels of parental income, p < 0.05 [11]; our study, by contrast, uses linear regression to account for individual variation in parental SES. In addition, rather than examining a single incident of trauma, we regress functional connectivity with the amygdala seed region on a self-report cumulative measure of childhood exposure to violence. Based on previous research, we expected childhood exposure to violence to predict decreased connectivity between the amygdala and the ACC. Moreover, we predicted decreased connectivity between the amygdala and the ACC based on the findings of a meta-analysis that concluded that peak effects of adversity across multiple studies converged at the ACC [34].

### Exposure to violence: effects on working memory circuitry

Many studies also show that childhood adversity, including physical and emotional abuse, is related to deficits in working memory, among other executive functions [20, 50–52]. Neuroimaging research also tracks with this behavioral evidence. The dlPFC, along with the dorsomedial prefrontal cortex (dmPFC) and inferior parietal cortex, comprise the frontoparietal network [53], which is involved in working memory and cognitive control [53, 54]. Several studies have demonstrated reduced connectivity in this network in relation to adversity [10, 49, 55, 56].

With regard to exposure to violence, individuals who experienced abuse during childhood compared to controls had reduced connectivity in the frontoparietal network during a sustained attention task [10]. Adolescents’ self-reported exposure to violence was also linked with reduced activity in frontoparietal regions during an executive control task [9]. While these studies have demonstrated decreased frontoparietal connectivity in relation to exposure to violence, other findings are mixed. One influential approach, the dimensional model of adversity and psychopathology [57], distinguishes between threat, defined as experiences of threat or harm, and deprivation exposures, defined as the absence of expected social, physical, and emotional environmental stimulation [57]. This model proposes that threat exposures including physical abuse, direct community violence, and family violence abuse, affect emotion regulation; while deprivation exposures including low income-to-needs ratio and parental education affect working memory [57, 58]. One study in line with the model found that deprivation (low parental education and childhood neglect), but not abuse was related to working memory deficits [55]. Overall, there is evidence that threat exposures including physical abuse, direct community violence, and family violence abuse impact emotion regulation; while deprivation exposures including low income-to-needs ratio and parental education impact working memory [57, 58]. However, further evidence is needed to test whether exposure to violence predicts decreased frontoparietal connectivity. Our study can help elucidate the specificity of exposure to community violence-related reductions in frontoparietal connectivity since we measured both parental SES and exposure to violence. Thus, we can assess the effects of Exposure to Violence after accounting for parental SES. However, since we are using SES and not directly measuring deprivation, we cannot draw conclusions about the effects of deprivation.

In the current study, we use a working memory-related region of the prefrontal cortex (the right dlPFC) as a seed to test for changes in connectivity with the rest of the brain as a function of exposure to violence. In line with previous research, we expect reduced connectivity between the right dlPFC seed and parietal regions involved in working memory, such as the intraparietal sulcus and surrounding cortex, for individuals with high compared to low childhood violence exposure. Alternatively, it is possible that we would find no differences in working memory circuitry based on exposure to violence and after accounting for SES.

### Cognitive control and emotion regulation

The two consequences of adversity considered here—emotion regulation and working memory—are distinct, yet related [59–62]. A meta-analysis, for example, identified overlap between working memory and emotion regulation networks, such as in the ACC, but also found distinct regions associated with each domain [44]. These interrelated domains are both broadly associated with adversity, yet the research is inconclusive as to whether each is independently associated with threat vs. deprivation exposures [23, 57].

In the current study, we examine emotion regulation and working memory networks, using the bilateral amygdala and dlPFC as seeds to test for differences in neural connectivity related to exposure to violence. We expect reduced connectivity in these two distinct but related networks, with both the right dlPFC and bilateral amygdala seeds showing reduced connectivity in medial prefrontal regions with increased exposure to violence. As mentioned previously, we also test for the possibility that after accounting for SES, exposure to violence will predict decreased connectivity in fronto-amygdala circuitry, but not in frontoparietal circuitry.

### Current study

Here we examine whether younger adults’ self-reported exposure to violence during childhood modulates intrinsic functional connectivity among emotion regulation and working memory circuits. Specifically, we examine the cumulative effects of exposure to violence during childhood and into early adolescence. We use functional magnetic resonance imaging (fMRI) to test for specific neural regions where connectivity differs based on exposure to violence in childhood.

To investigate areas relevant to these functions, in the current study we chose two seed regions from the amygdala and one seed from the right dorsolateral prefrontal cortex. We first conducted a group comparison of individuals exposed to high compared to low levels of violence. These groups were matched on current SES. A follow-up analysis was conducted on a subset of participants who had a more relevant measure of SES—parental SES. In this second analysis, exposure to violence and SES were entered as continuous predictors in a regression on resting state functional connectivity. This analysis was completed to confirm that our results demonstrate the specific effect of exposure to violence above and beyond childhood SES.

## Materials and methods

### Participants

Fifty-two right-handed native English speakers were recruited from the Rutgers University—Newark community using the following sources: the undergraduate participant test pool, the Rutgers graduate student listserv, Craigslist, social media groups, and flyers posted in several campus locations. Participants were combined across five separate studies conducted by the Language Behavior and Brain Imaging Laboratory, all of which collected resting state fMRI data in a separate run using identical acquisition parameters. All participants provided written informed consent according to Institutional Review Board guidelines and were compensated $30 per hour for their time in the scanner. Participants were prescreened to exclude those reporting a history of traumatic brain injury, psychiatric illness, diagnoses of learning disabilities, autism, or attention deficit hyperactivity disorder. Participants were also screened based on self-report for current drug use, cigarette smoking, and more than moderate alcohol consumption (more than 5 drinks per week).

To address our primary question of interest (connectivity differences related to exposure to violence), we selected two subgroups based on a median split of their self-report scores on an adapted version of the Survey of Exposure to Community Violence (SECV) [63]. They were selected for inclusion in one of two groups based on whether they reported being exposed to high levels of violence during childhood. To isolate the neural effects of previous exposure to violence, the two groups were selected to match on multiple relevant variables. The matching process began with the two initial groups (HighViol vs. LowViol). Continuous-valued variables were tested for differences between groups using between-subjects t-tests, while categorical variables were tested using chi-square tests. The two groups were initially matched on all variables except for race, χ^2^ (4, N = 52) = 12.36, p = 0.01, and current exposure to violence, t [50] = 2.41, p = 0.02. Participants were removed based on characteristics that would generate matched groups while maximizing sample size. The resulting sample was N = 46. The HighViol group consisted of 23 individuals, with 7 females; the LowViol group consisted of 23 individuals, with 13 females, χ^2^ (1, N = 46) = 02.21, p = 0.137. Based on assignment, groups differed significantly on self-reported exposure to violence during childhood, High Violence (M = 20.30, SD = 5.97), Low Violence (M = 6.0, SD = 3.10), t [33] = 10.19, p < 0.0001. These are unitless numbers based on a self-report scale, as described further below. After matching, there were no reliable differences between groups for the continuous-valued variables of current SES, age, and exposure to violence during the past year (all p > 0.1, Table 1). Groups were also matched on race, χ^2^ (3, N = 46) = 6.83, ethnicity, χ^2^ (2, N = 46) = 3.09, and languages spoken (monolingual vs. bilingual), χ^2^ (1, N = 46) = 0.57 (all p > 0.05, Table 1). Language data for two participants were missing. To address this issue, the data were imputed with all possible combinations of language for these two participants; it was found that regardless of whether those two participants were monolingual or bilingual, language would still be matched between groups, all p > 0.6. Finally, a power analysis in R [64] using alpha = 0.05 and power = 0.80 demonstrated that the sample size (N = 46) was sufficient to detect a medium to large effect (Cohen’s d > 0.8).

**Table 1:** Characteristics for participants included in the group comparison

	High Violence	Low Violence	P-value
N = 46	23	23	
Age	23.70	22.57	0.22
Gender	7 Females	13 Females	0.14
SES current	16.26 (4.58)	16.26 (3.98)	1
SES parental	N = 12 12.42 (4.25)	N = 9 13.33 (4.80)	0.65
Exp Viol Current	8.35 (6.19)	5.43 (4.45)	0.07
Exp Viol Childhood	20.30 (5.97)	6.00 (3.10)	< 0.001
Languages Spoken (Monolingual vs. bilingual)	16 monolingual 5 bilingual Missing: 2	14 monolingual 9 bilingual	0.44
Race	1 Asian 11 Black 6 White 1 Other	**7** Asian 7 Black 11 White 1 Other	0.08
Ethnicity	8 Hispanic-Latino 15 Not Hispanic-Latino	2 Hispanic-Latino 21 Non Hispanic-Latino	0.07

Individuals scoring above the median (High Violence) on the adapted version of the Survey of Exposure to Community Violence (SECV) [63] were matched to individuals scoring below the median (Low Violencnce) on the SECV for the listed variables

For our secondary question regarding the separability of these effects from SES, we used a subset of the full sample that also had parental SES and conducted a linear regression analysis. Parental SES scores were only available for a subset because after approximately half of participants were recruited, it was determined that a more specific measure of parental SES, rather than current SES, would be helpful in identifying the effects of childhood exposure to violence above and beyond childhood SES. As such, a measure of parental SES was collected in a subset of participants (N = 25), of the original full sample (N = 52). For this group, the correlation between parental SES and current SES was 0.41, p = 0.04. In this sample, the average self-reported exposure to violence during childhood was 15 (SD = 10.47), exposure to violence during the past year was 7.92 (SD = 9.21), the average age was 21.72 (SD = 2.57); 5 participants (20%) were female. A power analysis in R [64] using alpha = 0.05 and power = 0.80 demonstrated that the sample size (N = 25) was sufficient to detect medium to large effect (Cohen’s f^2^ > 0.35).

### Procedure

Resting-state data were collected at the end of each scanning session (after completion of any experimental tasks). Participants were told to lie still, look at the fixation cross, and let their mind wander. For three of the studies, participants completed relevant questionnaires directly after the brain scan. For the other three studies, participants were contacted after they had participated in the study, and invited to complete the questionnaires online.

### Behavioral measures

The Survey of Exposure to Community Violence (Richters & Saltzman [63]) is a validated self-report measure that assesses an individual’s incidence of being a victim of violence (e.g., Have you been hit or punched by someone?) as well as witnessing acts of violence (e.g., Have you heard guns being shot?). The full survey, which consists of 11 items assessing victimization and 35 assessing witnessing violence, has high test–retest reliability (0.81, Richters & Martinez [65]). In the current study, we selected a subset of the most relevant items using an approach similar to Boxer et al. [38]. Two items assessed frequency of being a victim, and 13 items assessed frequency of witnessing violence. Four response choices are included for each question (1 Yes, many times; 2 Yes, a few times; 3 Yes, once or twice; 4 No.) Responses were reverse coded, with choice 4 receiving 0 points, and choice 1 receiving 3 points, etc. This coding scheme was used so that higher numbers could be straightforwardly interpreted as more exposure to violence. Two items asking about helping behavior (e.g., Have other people helped you with something?) were not scored. Two copies of the survey were administered to all participants. For one, participants were asked to base their answers on their childhood experience during the ages of 3 to 16 years old. Scores on this measure (Childhood exposure to violence) were used as the main predictor in our analyses. To control for current exposure to violence in the group comparison, a second copy of the survey was administered, for which participants were asked to base their answers on their current experiences, during the last year.

The Modified Kuppuswamy SES scale was adapted from Kuppuswamy [66]. It is a three-item measure assessing education and occupation of the head of household, as well as per capita income. Seven response choices were provided for each question, with responses indicating high SES (e.g. “Professional”) coded as 7, and low SES (e.g. “Unemployed”) coded as 1. Thus, scores range from 3 to 21, with lower scores indicating lower SES (labeled “SES current” in Table 1). After administering this survey to twenty participants, it was determined that it would be additionally useful to obtain a measure of childhood SES, as described above. Therefore, an additional measure was administered based on the Reserve Capacity Model [67]. It asked participants to report household income, employment status, and highest level of educational attainment of their parent or guardian (labeled “SES parental” in Table 1). Twenty-five participants of the full sample (N = 52) completed it. The correlation between parental SES and childhood exposure to violence among the 25 participants who completed it was − 0.34, p = 0.09.

### Seed ROI generation

**Fig. 1:**
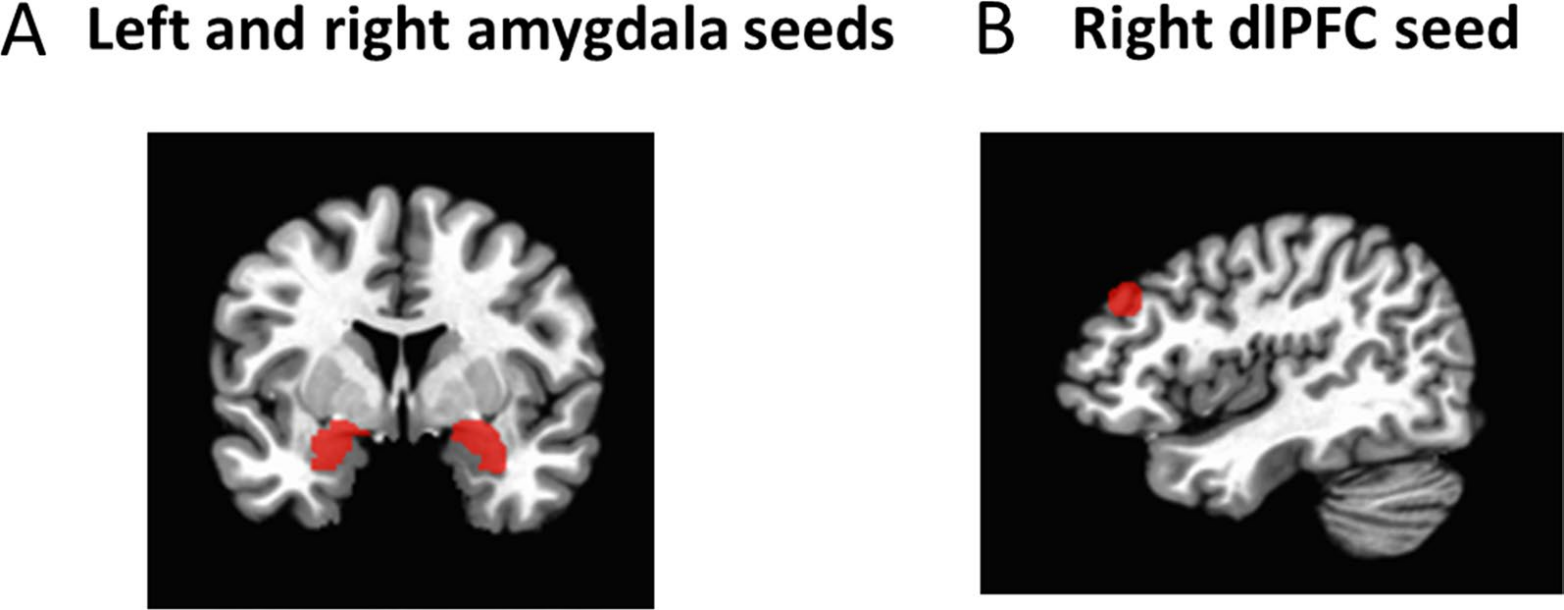
Seed regions. **A** Left and right amygdala seeds. **B** Right dorsolateral prefrontal cortex (dlPFC) seed (centered at Talairach coordinates, x = 43, y = 33, z = 31)

Bilateral amygdala ROIs (see Fig. 1A) were obtained based on anatomical designations from the Talairach atlas [68]. The right dlPFC was chosen as an additional seed based on evidence of adversity-related differences in connectivity [69], recruitment, [70] and gray matter differences [71] in the right dlPFC. Additionally, a focus of our original research was to explore the role of adversity-related working memory deficits in learning from negative feedback, which led us to choose a right dlPFC seed from a feedback learning study [72]. In this study, recruitment of the right dlPFC was involved in learning from negative feedback, with involvement attributed to working memory processes [72], which is relevant to our current research question. This right dlPFC ROI (Fig. 1B) was generated by creating a 6 mm sphere centered on the coordinates from this study [72] and converted from MNI to Talairach space as x = 43, y = 33, z = 31 [73, 74]. This resulting ROI is part of the middle frontal gyrus and has been shown to be involved in working memory across several meta-analyses [75–77], thus confirming its role in working memory. Notably, the ROI is essentially identical with the highest-peak right dlPFC result (x = 40, y = 32, z = 30) from an influential meta-analysis of fMRI studies on working memory (79; See Additional file 1: Fig. S4 for results using the ROI from this meta-analysis).

After the three seed ROIs were generated, they were aligned to each subject's original resting state functional image space by inverting the transformation matrix already calculated for registering the individual images to group space.

### fMRI acquisition and analysis

MRI data were collected on a 3-T Siemens Magnetom TrioTim Scanner with a 12 channel head coil. A T1 high-resolution anatomical brain scan was collected for each participant, using a 3-dimensional magnetization-prepared rapid gradient-echo (MPRAGE) sequence, with a TR of 1900 ms (ms) and a TE of 2.52 ms (matrix = 256 × 256 voxels, 176 contiguous 1 mm axial slices, field of view, FOV = 256 mm, flip angle = 9 degrees). A seven-minute resting state scan of Blood Oxygen Level Dependent (BOLD) data was collected using a gradient-echo echoplanar imaging (EPI) sequence (TR = 3000 ms, TE = 31 ms, FOV = 240 mm, matrix = 96 × 96 voxels, flip angle = 90 degrees), yielding 140 TRs and 41 oblique axial slices roughly parallel to the anterior commissure-posterior commissure plane with 2.5 × 2.5 × 2.5 mm voxel size.

All MRI data were preprocessed using the AFNI software suite (http://afni.nimh.nih.gov/afni; [73]. Slice timing and motion correction were applied to the time series images, and the high-resolution structural scan was then aligned to these images [78]. The first 6 images in each run were ignored due to initial saturation. High and low pass filtering between 0.01 and 0.1 Hz was applied to the image time series. Signal in the ventricles and white matter, as well as the global mean, were modeled separately as nuisance regressors using the AFNI program for least squares multiple linear regression, 3dDeconvolve. The time series for each voxel in a given seed ROI was averaged to create a single mean time series for that ROI using the AFNI program 3dmaskave, and entered into an individual subject whole-brain analysis as the regressor of interest for each subject. Each subject’s anatomical scan was aligned to the Talairach atlas [79], and this alignment solution was applied to register each subject’s image regression results to the same atlas.

Group-level analyses were performed with a two-sample *t*-test, using AFNI’s 3dttest +  + , for each functional connectivity map of each ROI by comparing between the groups High Exposure to Violence vs. Low Exposure to Violence (n = 46). These groups were matched on current SES. As a follow-up, we complete an analysis with exposure to violence as a continuous factor while also including childhood SES as a control covariate (which was only available in a subset of participants, n = 25). In this analysis, parental SES and Childhood Exposure to Violence were entered as predictors into the 3dttest +  + function in AFNI.

A brain mask excluding most white matter and cerebrospinal fluid was applied to all resulting images. The group z-score images were thresholded at a voxelwise p < 0.005, with a cluster extent correction of 532 mm^3^ (mapwise corrected p < 0.05, as determined by the AFNI program 3dClustSim). Analyses were performed separately for the amygdala and dlPFC seed regions.

To extract beta-values (connectivity values) from the clusters for purposes of plotting, the 3dmerge command was used to generate thresholded, cluster-corrected image results of each analysis. The 3dcalc command was then used to extract specific clusters that were significant in the relevant whole-brain connectivity analysis, such as the region of the ACC that showed reduced connectivity in relation to higher childhood exposure to violence. Finally, the 3dROIstats command was used to extract the mean connectivity value for each subject within the region corresponding to the cluster.

A conjunction analysis was conducted to determine the areas of overlap between the results of the right dlPFC and right amygdala resting state connectivity analyses [80]. The conjunction analysis was run by using the 3dmerge and 3dcalc commands in AFNI to generate two binary maps of the region of the ACC that resulted from the regression analysis using the right dlPFC seed and the right amygdala seed. The 3dcalc command was then used to multiply these images by each other, such that only areas of overlap remained.

## Results

### Amygdala resting state functional connectivity

#### Group comparison: amygdala seeds

First, we established the overall connectivity patterns for each group, then contrasted the connectivity between groups. For both groups, the analysis examining resting state functional connectivity of the amygdala seed regions with the whole brain resulted in positive connectivity with numerous regions throughout the brain, not qualitatively different for the left and right seeds. These included the medial and orbital frontal cortex, areas of dorsal frontal cortex (superior frontal gyrus), bilateral amygdala, parahippocampal gyrus, caudate, temporal lobe, aspects of both the inferior and superior parietal lobe, posterior cingulate, insula, and bilateral occipital cortices (See Additional file 1: Fig. S1).

**Fig. 2:**
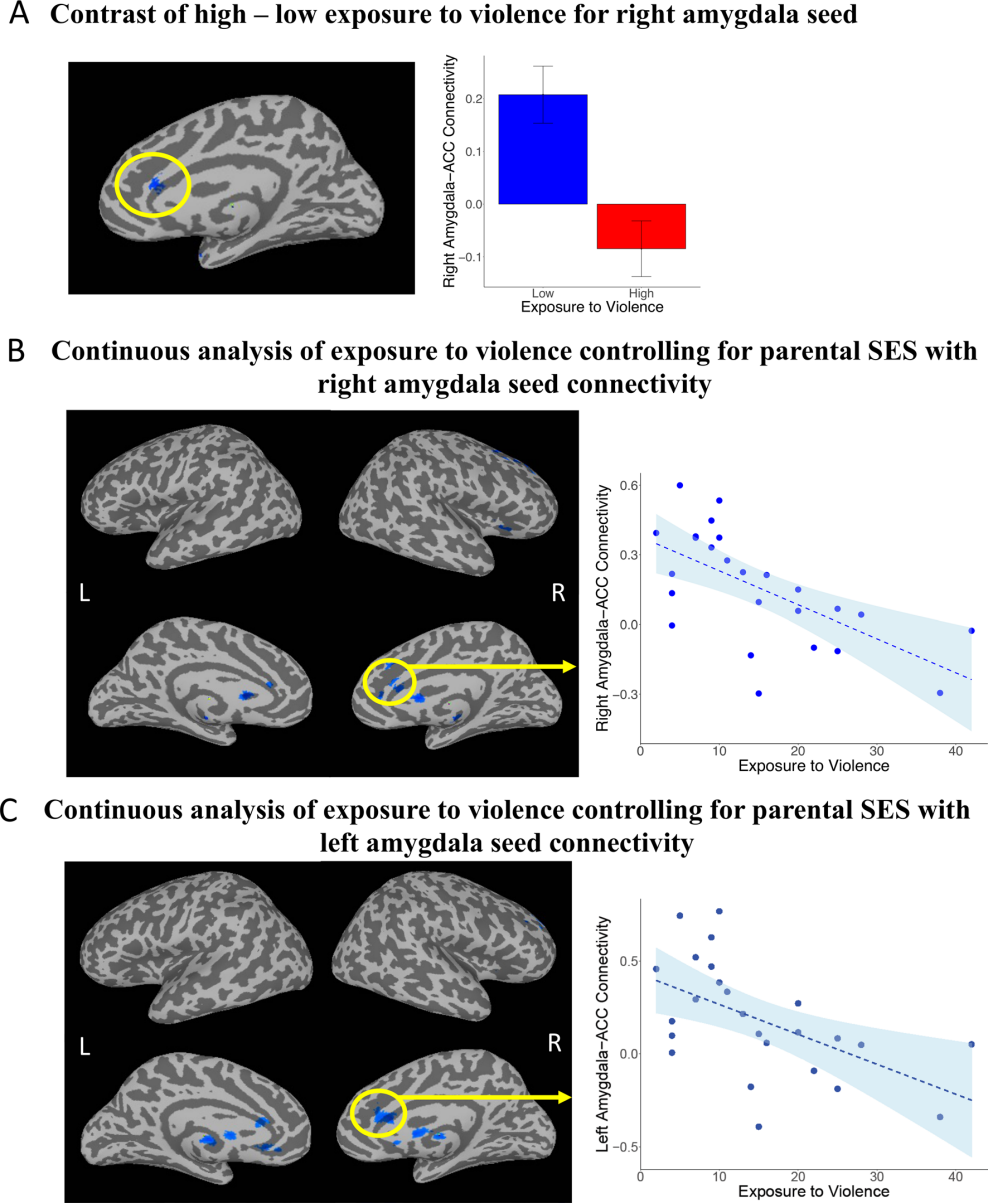
Functional connectivity results from the right amygdala seed. **A** The contrast of resting state functional connectivity with the right amygdala seed for High Violence—Low Violence. **B** Continuous analysis of exposure to violence controlling for parental SES and with right amygdala seed connectivity**. C** Continuous analysis of exposure to violence controlling for parental SES and with left amygdala seed connectivity

The contrast of high minus low exposure to violence was expected to show weaker connectivity for the high exposure to violence group between the amygdala seeds and frontal regions involved in cognitive control of emotion, such as the ACC. In line with this hypothesis, the contrast yielded significantly less connectivity for high- compared to low-violence exposure between the right amygdala and the ACC (Fig. 2A, Table 2). The contrast of functional connectivity with the left amygdala, however, showed no significant difference between groups.

**Table 2:** Talairach coordinates of the resting state functional connectivity analyses with the amygdala seeds

Location of extreme point	Cluster size (µl)	x	y	z	Peak z statistic
Contrast of high—low exposure to violence for right amygdala seed					
*HighViol* > *LowViol*					
No significant activations					
*LowViol* > *HighViol*					
Right temporal pole	866	26	− 15	− 18	3.57
Left temporal cortex	738				
L inferior temporal gyrus		− 56	− 20	− 18	4.19
L parahippocampal gyrus		− 28	− 2	− 26	3.68
Anterior cingulate	552	1	32	17	3.58
Left putamen	538	− 20	− 8	11	4.11
Continuous analysis of exposure to violence controlling for parental SES with right amygdala seed connectivity					
Increased connectivity with increased violence scores:					
No significant activations					
Decreased connectivity with increased violence scores:					
Left red nucleus	1249	− 6	− 20	− 9	4.14
Right anterior cingulate	1149	5	27	19	3.35
Right middle frontal gyrus	1079	23	5	38	4.30
Cerebellum	861	1	− 58	− 31	4.04
Right caudate	788	1	10	11	3.82
Right insula	704	31	13	3	3.75
Right superior frontal gyrus	608	23	32	33	3.60
Left cerebellum	591	− 35	− 55	− 26	3.54
Continuous analysis of exposure to violence controlling for parental SES with left amygdala seed connectivity					
Increased connectivity with increased violence scores:					
Cerebellum	715				
		24	− 30	− 48	3.54
		− 25	− 69	− 43	3.51
Decreased connectivity with increased violence scores:					
Right caudate	4220	8	1	6	5.02
Right anterior cingulate cortex	1867	15	32	7	4.09
Anterior cingulate cortex	1180	4	26	19	3.79
Right superior frontal gyrus	918	26	50	30	3.83
Left cerebellum	874	− 16	− 56	− 31	4.93
Cerebellum	662	1	− 43	− 30	4.23

#### Continuous analysis: amygdala seed

The analysis treating exposure to violence as a continuous variable, statistically controlling for parental SES, showed similar results to the categorical analysis above. Increasing levels of exposure to violence significantly predicted decreasing connectivity between the right amygdala and the ACC, (see Fig. 2B, Table 2). Results were similar with the left amygdala seed, where higher exposure to violence predicted lower connectivity with the ACC, bilateral putamen, and right dlPFC (see Fig. 2C, Table 2).

A post-hoc resting state connectivity analysis was conducted on the entire sample (N = 52) and using the same amygdala seeds, but with current, instead of parental, SES entered as a covariate. As in the main regression analysis, exposure to violence was entered as the predictor variable. This analysis showed that after accounting for current SES, increasing exposure to violence predicted decreased connectivity between the left amygdala and thalamus (Additional file 1: Fig. S2A), and between the right amygdala and the left anterior middle temporal gyrus (Additional file 1: Fig. S2B).

### Right dorsolateral PFC seed resting state functional connectivity

#### Group comparison: right dlPFC seed

For both the groups, exposed to low and high levels of violence, the analysis examining resting state functional connectivity between the right dlPFC seed region and the whole brain resulted in positive connectivity with the lateral, dorsolateral, and dorsomedial frontal cortex, insula, thalamus, putamen, precuneus, bilateral inferior and superior parietal lobules, and bilateral occipital lobe.

**Fig. 3:**
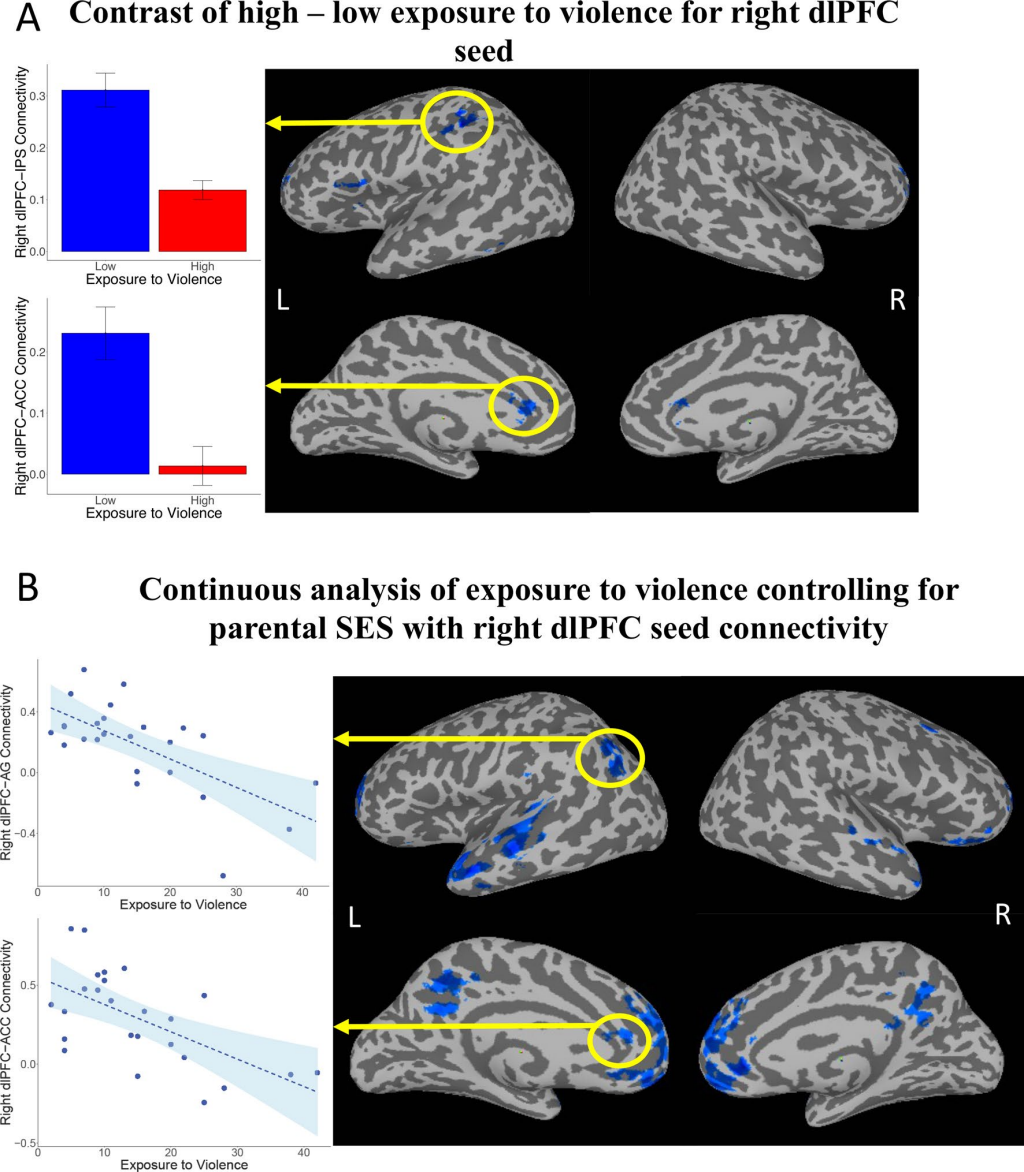
Functional connectivity results from the right dlPFC seed. **A** The contrast of resting state functional connectivity with the right dlPFC seed for High Violence—Low Violence. **B** The results of the continuous analysis using the right dlPFC seed, regressed on Exposure to Violence scores

The contrast of high minus low exposure to violence revealed significant decreased connectivity between the right dlPFC seed and the cortex surrounding the left IPS, left inferior frontal gyrus, ACC, bilateral vlPFC, and left insula (see Fig. 3A, Table 3).

**Table 3:** Talairach coordinates of the resting state functional connectivity analyses with the right dlPFC seed

Location of extreme point	Cluster size (µl)	x	y	z	Peak z statistic
Contrast of high—low exposure to violence for right dlPFC seed					
HighViol > LowViol					
No significant activations					
LowViol > HighViol					
Left middle frontal gyrus	2537	− 15	41	15	3.80
Left inferior parietal sulcus	1926	− 42	− 31	39	4.35
Right superior frontal gyrus	897	23	50	7	3.80
Right anterior cingulate cortex	847	13	15	24	4.53
Left caudate	634	− 19	20	8	3.04
Right cerebellum	571	35	− 50	− 35	4.43
Left inferior temporal gyrus	552	− 45	− 37	− 10	3.79
Continuous analysis of exposure to violence controlling for parental SES with right dlPFC seed connectivity					
Increased connectivity for increasing violence					
No significant activations					
Decreased connectivity for decreasing violence					
Frontal cortex	22,445				
Orbitofrontal cortex		0	59	− 9	4.69
R middle frontal gyrus/Ventrolateral PFC		27	51	13	4.57
L middle frontal gyrus/Ventrolateral PFC		− 29	53	12	4.53
Left precuneus	5484	− 1	− 47	34	3.95
Left middle temporal gyrus	4712	− 45	− 6	− 15	4.74
Right superior temporal gyrus	3992				
R superior temporal gyrus/R temporal pole		44	22	− 18	4.11
R superior temporal gyrus		56	− 16	− 3	3.42
Left middle temporal gyrus	2638	− 54	− 21	1	4.04
Left angular gyrus	2284	− 38	− 59	33	4.55
Right middle frontal gyrus	725	32	17	47	3.79
Left caudate	587	− 22	3	24	− 3.90

#### Continuous analysis: right dlPFC seed

The continuous analysis with the right dlPFC seed, statistically controlling for parental SES, yielded results largely compatible with but more spatially extensive then the discrete group analysis. With increasing reported childhood exposure to violence there was decreasing connectivity between the right dlPFC and the ACC, vmPFC, left angular gyrus, left caudate, and the bilateral anterior temporal lobe (see Fig. 3B, Table 3).

As with the amygdala seeds, another post-hoc regression analysis was conducted using the right dlPFC seed, on the entire sample (N = 52), with current SES as a covariate instead of parental SES. This analysis showed that after accounting for current SES, increasing exposure to violence predicted decreased connectivity between the right dlPFC seed and the bilateral anterior middle frontal gyrus and bilateral IPS (see Additional file 1: Fig. S3).

### Conjunction analysis

**Fig. 4:**
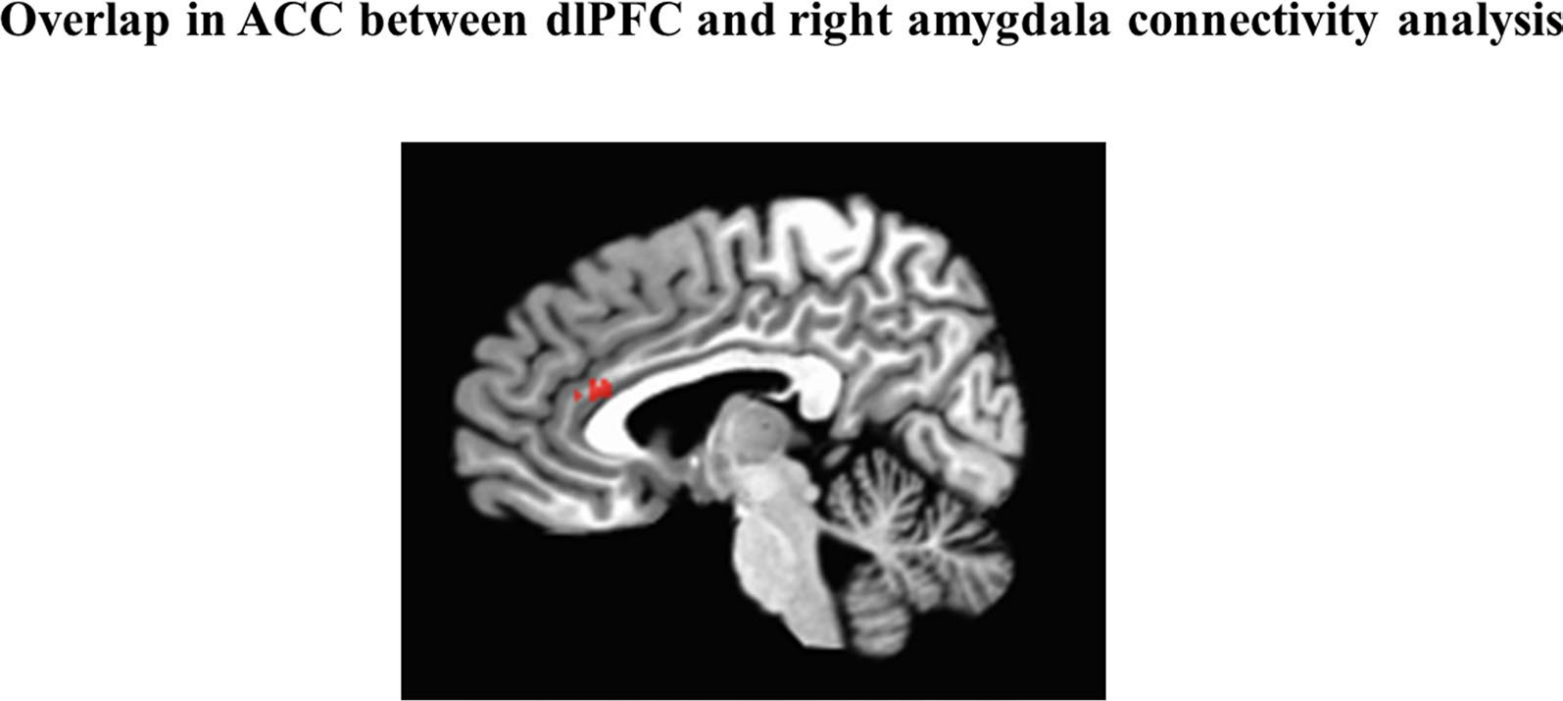
Results of the overlap analysis. This region of the ACC showed reduced connectivity to the right dlPFC seed and the right amygdala seed in relation to high childhood exposure to violence

In viewing the results of the continuous analyses from the right dlPFC and amygdala seeds, it appeared that a region of the ACC showed reduced connectivity with both the right dlPFC and the right amygdala seeds with higher reported childhood exposure to violence. To confirm this, a conjunction analysis [80] was run to determine whether there was spatial overlap. We binarized the map for the two resulting maps of the two seed regions and determined their intersection. This resulted in overlap region of 159 of voxels in the ACC (see Fig. 4).

## Discussion

Childhood adversity, including exposure to abuse, history of trauma, and deprivation of typical environmental experiences, has been shown to be related to differences in regulation of affect and cognition [20, 23, 26, 81, 82]. The results of the current study suggest that, after controlling for SES, exposure to violence during development is specifically related to differences in neural circuits associated with emotion regulation and cognitive control. In particular, participants reporting high, compared to low, levels of exposure to violence during childhood demonstrated less functional connectivity between amygdala and prefrontal regions, particularly the ACC, implicated in emotion regulation [34, 47]. In a different analysis that specifically accounted for parental SES, higher reported violence predicted less connectivity between the amygdala and prefrontal regions such as the ACC. The results of our right dlPFC connectivity analysis were less clear. While the group comparison demonstrated decreased connectivity between the right dlPFC and intraparietal sulcus, our second analysis, which accounted specifically for parental SES, showed decreased connectivity between the right dlPFC and a different parietal region, the angular gyrus (AG). Our results suggest that exposure to violence (seeing and hearing about violent acts) during development, after accounting for SES, is related to differences in adult neural circuitry relevant to both affective and cognitive functioning.

### Amygdala connectivity

Our current finding of decreased connectivity between the right amygdala and the rostral ACC for individuals exposed to high compared to low levels of violence is consistent with previous evidence of reduced prefrontal-subcortical connectivity resulting from acute [83] and chronic stress [11, 22, 84, 85]. It is also in line with adversity-induced increases in sensitivity to threat [56, 86, 87], which is thought to be related to decreased regulation of subcortical regions, like the amygdala, by prefrontal control regions [24, 25, 34, 88–92]. Our finding of decreased connectivity between amygdala and rostral ACC for individuals exposed to high levels of violence is also consistent with structural brain findings, such as adversity-related lower integrity of white matter tracts connecting frontal with medial temporal regions [93, 94] and reduced gray matter volume in the frontal cortex among children exposed to domestic violence [95].

The results of the continuous analysis on the subset of participants with parental SES scores substantiates these findings and lends additional support to the conclusion that exposure to violence predicts reduced amygdala-ACC connectivity, above and beyond the effects of SES.

Therefore, while both low parental SES and exposure to violence represent subtypes of adversity, the contribution of the current study is to begin to decompose the construct of adversity into components, showing that early exposure to violence has an independent impact on emotion regulation circuitry. Further, reduced connectivity with higher reported violence exposure offers evidence of a continuous, compounding effect of chronic exposure to violence rather than an “all-or-nothing” type effect related to an exposure threshold.

Finally, our findings are also consistent with a meta-analysis demonstrating that decreased connectivity between the amygdala and the rostral ACC, rather than the vmPFC, is related to adversity [34]. Our study contributes to the literature in that it offers additional evidence of the specificity of the role of the ACC in stress-induced decreases in fronto-amygdala connectivity.

### Dorso-lateral prefrontal cortex connectivity

The contrast of resting state functional connectivity maps between the high compared to low violence exposure groups showed decreased connectivity between the right dlPFC seed and the bilateral IPS, left inferior frontal gyrus, ACC, bilateral vlPFC, and left insula. The dlPFC and IPS have previously been shown to be involved in working memory [75, 96] among other cognitively demanding tasks [97]. While our group analysis showed reduced right dlPFC-IPS connectivity for the group exposed to high violence, the continuous analysis on the subset of participants with parental SES scores (N = 25) revealed a somewhat different pattern. Specifically, after accounting for SES in the continuous analysis, the right dlPFC no longer showed reduced connectivity with the IPS for higher exposure to violence. Thus, standard working memory circuitry was not related to exposure to violence after accounting for SES.

Several studies show working memory deficits and reduced frontoparietal connectivity related to adversity [69, 87, 98, 99]. However, the dimensional model of adversity and psychopathology suggests that working memory deficits may be specific to deprivation-related adversity, but not threat-related adversity [58, 100]. The results of our dlPFC connectivity analysis lend support to this, as dlPFC-IPS connectivity was no longer significant after accounting for SES.

In addition to stress-induced decreases in frontoparietal connectivity, adversity-related differences have also been shown across frontal regions [26, 40, 100]. In fact, a meta-analysis found decreased volume in frontal regions across several subtypes of adversity, including sexual abuse, emotional maltreatment, and neglect [101]. Another meta-analysis found reduced grey matter volume in the right dlPFC as well as the amygdala among those who had experienced childhood trauma [71]. Such reductions in dlPFC volume may underlie reduced connectivity [102]. Overall, the results of our dlPFC resting state connectivity analysis are in line with this research, as both the group comparison and continuous analysis demonstrated reductions in connectivity across frontal regions for higher reported childhood exposure to violence.

In the continuous analysis, decreased dlPFC—AG, but not dlPFC—IPS, functional connectivity was found with increasing levels of exposure to violence after accounting for a more precise measure of SES—parental SES. While this finding was not predicted, it is consistent with another study showing that exposure to violence predicted reduced activity in a region outside of IPS, but within the posterior parietal lobe during an inhibition task among adolescents [9]. More broadly, it is also in line with literature on emotion regulation circuitry. Since childhood adversity has been shown to predict emotion regulation [11, 89], such findings seem relevant here. One study, for example, found decreased gyrification, shown to underly connectivity differences [103], in the AG for participants with emotion regulation difficulties [104]. Another meta-analysis suggests that the dlPFC is involved in regulation of emotion and the AG in execution of emotion regulation [16]. The results of our regression analysis are in line with research showing that violence exposure, but not poverty, was associated with emotion regulation difficulties; and poverty, but not violence, is associated with cognitive control deficits [105]. Moreover, both forms of adversity were related to decreased inhibition under emotional conditions [105]. Thus, while decreasing dlPFC-AG connectivity for higher exposure to violence was not predicted, it lends support to the proposition that exposure to violence alters connectivity in emotion regulation circuitry—fronto-amygdala and frontal-AG—but not working memory itself (fronto-IPS). Such results warrant further attention to distinct aspects of the parietal lobe in follow-up studies.

Finally, in both the grouped comparison and the continuous analysis, the dlPFC showed less connectivity with the ACC with increasing levels of exposure to violence. Previous research has shown that the dlPFC mediates the relationship between the ACC and the amygdala [30, 106], and thus is relevant to the emotion regulation circuit. The results of our connectivity analysis with the dlPFC seed suggest that while exposure to violence may not account for decreased connectivity in working memory circuitry, it may account for decreased connectivity in a broad network associated with emotion regulation.

### Amygdala and dlPFC connectivity differences converge in the ACC

Both the right amygdala and right dlPFC seed connectivity analyses showed reduced connectivity with the ACC—with a spatial overlap between the analyses. The right dlPFC seed was chosen because of its role in working memory, and the right amygdala for its association with the threat response. Results of our connectivity analyses in the ACC point to it being at the intersection of cognitive and emotional control. In line with this interpretation, the ACC has been implicated in the regulation of both cognitive and emotional processing [47, 107–109]. Moreover, the ACC has been shown to influence limbic regions [48, 110–112], and to be involved in the appraisal and regulation of negative emotions [111]. Other related work suggests that emotion regulation depends upon the interaction between prefrontal—cingulate and cortical—subcortical circuitry [48, 113].

The results of our analyses are in line with this proposal and offer evidence that dlPFC-ACC connectivity may be related to decreased amygdala-ACC connectivity. Importantly, since SES was accounted for in the continuous analysis, this study offers evidence that both cortical and subcortical circuitry differences are specific to threat exposure above and beyond other effects of SES. Overall, while the results do not support threat-related differences in working memory circuitry, they do support threat-related differences across frontal regions, which may in turn be related to the emotion regulation circuitry. However, considering our analyses included resting state data without corresponding affective or cognitive measures, we cannot interpret results as specifically relating to a particular affective or cognitive process. Moreover, various functions are associated with the ACC, including effortful and attentional control [16, 114], conflict monitoring [115], error detection [47, 116], and emotion regulation [16, 34, 47, 117]; and thus, a single process cannot be assumed to be related to connectivity differences. However, given impaired emotion regulation among individuals affected by threat exposures [11, 39, 40, 58, 118]; relationship between ACC and adversity [34, 119, 120]; and evidence for the role of the ACC in the processing of negative emotion and effects on limbic regions [111], it is plausible that adversity-related differential connectivity between the ACC and amygdala would correspond to cognitive regulation of emotion.

### Threat vs. deprivation exposure

Our continuous analysis with parental SES as a covariate confirmed that decreased right amygdala-ACC connectivity is associated with childhood exposure to violence even after accounting for SES. These results are consistent with the dimensional model of adversity (cite 53,95), which suggests that threat exposure but not deprivation *per* se is related to decreased right Amygdala-ACC connectivity. However, while deprivation is a broad construct related to the absence of expected environmental input [57], we measured SES, which may or may not capture some of the individual variation in deprivation exposure. Specifically, our measure of parental SES was comprised of parental income, education, and employment. Other sources of deprivation that could have been measured include neglect. Additionally, it is possible for an individual to report low parental SES, but to have had sufficient supports (community, extended family, etc.) such that low SES did not correspond to deprivation. As such, parental SES is not an explicit measure of deprivation and thus we cannot draw conclusions about the effects of deprivation on neural connectivity. Given the lack of an explicit measure of deprivation, caution should also be taken when interpreting our results as being uniquely related to threat exposures. Thus, although our results are seemingly consistent with the dimensional model of adversity and psychopathology [58, 82], further studies are needed to substantiate this claim. What we can state is that our study offers evidence that exposure to violence affects right amygdala-ACC connectivity above and beyond the effects of parental income, education, and employment.

Lastly, our post-hoc analyses accounting for current, instead of parental SES, might lend support to future studies exploring the role of current SES in moderating effects of childhood exposure to violence. Specifically, analyses of current SES using the amygdalae as seed regions (Additional file 1: Fig. S2) showed essentially null results with respect to our hypotheses, engaging no frontal lobe circuitry. The results of the analysis with the right dlPFC seed and current SES as a covariate were consistent with the results of our discrete contrast analysis between high and low exposure to violence groups, which matched groups for SES overall, without specifically distinguishing current from parental SES. Both results showed that increased exposure to violence predicting decreased right dlPFC—left IPS connectivity. Caution is warranted in interpreting this apparently convergent result, however, in that the discrete contrast result (Fig. 3A) showed anterior IPS connectivity with right dlPFC, while the continuous analysis of current SES (Additional file 1: Fig. S3) showed a more posterior IPS result, with additional connectivity in the right IPS. The current SES analyses should also be interpreted with caution given difficulties in measuring SES among college students [121]. They do, however, lend support to future studies exploring mitigation of childhood exposure to violence effects by current SES. With regards to the dimensional model of adversity [58, 82], future studies might aim to pinpoint effects of timing of threat and deprivation exposures.

### Implications

Here we have shown that individuals exposed to high levels of violence during childhood have decreased connectivity between the right amygdala and the ACC. In previous studies, decreased connectivity between ACC and the amygdala has been shown to be related to impairments in regulation of threat response [35, 91, 92]. Thus, if individuals affected by adversity have less connectivity between the ACC and the right amygdala, they may also be less able to regulate attention and emotional reactions to negative or threatening information.

Previous studies have operationalized adversity as having experienced childhood trauma, such as physical or emotional abuse; or having experienced deprivation related to, for example, being raised in an institution [21, 26, 43, 57, 82, 122–125]. Others have looked at effects of SES [17]. Our study, however, is unique in that we examine brain differences related to self-reported childhood exposure to violence. In the Exposure to Community Violence survey we administered, 13 out of 15 of the items corresponded to witnessing violent events, such as seeing someone shot with a firearm, seeing people with guns or knives in one’s neighborhood, seeing people be hit or pushed, and seeing people break windows on cars or buildings. Only two items related to being a victim (Have you been hit or pushed by someone? Have other people threatened to hurt you?). With composite scores generated by adding responses to the 15 items, it is inevitable that scores largely represent exposure to, rather than being a victim of, violence. Although the two constructs are likely correlated with each other, our study offers evidence that decreased frontal and fronto-limbic connectivity may be related to “mere” exposure to violence. Moreover, our study confirms that such differences in brain connectivity hold even after accounting for parental SES.

The lack of significant relationship between exposure to violence and connectivity between dlPFC and IPS, after accounting for SES, suggests that working memory deficits may not be related to exposure to violence per se, but may be related to other factors that commonly coincide with such exposure. The results of the conjunction analysis suggest that interventions targeting dlPFC-ACC connectivity may serve to buffer against effects of violence exposure on fronto-limbic circuitry. This is in line with previous studies showing, for example, that real-time fMRI neurofeedback was effective in increasing dlPFC-ACC connectivity and reducing anxiety for highly anxious individuals [126]. Viewed in light of the current results, this neurofeedback intervention may also be promising for individuals affected by exposure to violence.

### Limitations

This study offers insight into the neural connectivity associated with exposure to violence. We are, however, aware of several limitations. For one, exposure to violence is typically higher in neighborhoods of lower SES [127], and SES has been shown to correlate with multiple other environmental factors, such as exposure to environmental toxins [128]. Although SES was matched in our study, it is possible that other factors which co-occur with SES may also have differed between the groups. Exposure to violence may also affect other outcomes, such as level of physical activity [129], and perceptions of social cohesion among neighbors [127]. As such, it may be that differences in connectivity between groups is attributable to other violence- or low SES-related factors such as neglect. Exposure to violence may also be correlated with drug use and addiction [130]. However, in our sample, participants were prescreened based on self-report for no previous drug or alcohol abuse treatment, no current use of recreational drugs, and minimal alcohol consumption and/or cigarette smoking.

There were additional behavioral measures of potential interest, such as perceived stress, working memory abilities, and aggressive behaviors, that were not measured in this study. Thus, it cannot be concluded that the effects of exposure to violence are the same as, or different from, the effects of these variables. Further, the adapted version of the SECV [63] used in our study does not distinguish between exposure to family or partner violence compared to neighborhood violence. Therefore, the effects of exposure to violence in this study cannot be attributed to those more specific forms of violence.

Additionally, the number of participants with parental SES scores would ideally be larger; however, the regression analysis on this subset of participants was conducted as a secondary, follow-up analysis to the group comparison. Further, previous studies have found effects using a regression analysis with comparable sample sizes [132, 133]. Moreover, overlap between the results of the group comparison (N = 46) and the regression analysis (N = 25) substantiate our main findings. The discrepancy in results between the two analyses (in the group comparison, the IPS was significantly less connected to the right dlPFC; in the regression analysis, the AG was significantly less connected to the right dlPFC) should be interpreted with caution. These findings, nevertheless, call for further research to examine distinctions in frontoparietal connectivity in relation to exposure to violence and SES.

A final caveat is that this study of course cannot assert a causal relationship between exposure to violence and neural connectivity, as it relied on self-report data and was not an intervention study. This leaves the possibility that some participants may have inaccurately recalled exposure to violence during their childhood years. However, the results of the current study do converge with previous experimental work examining the effects on neural activation of acute stress. For example, participants who received a stress manipulation, compared to control participants, had increased activity in the amygdala in response to both threatening and positively valenced facial expressions [134]. These convergent findings point to the validity of the current results. If experiences of acute stress result in increased neural responsivity to threat and reward, with corresponding decreased PFC activity, it is logical that after prolonged exposure to stressors, the intrinsic functional connections between PFC control regions and affective subcortical and adjacent cortical regions would be reduced.

## Conclusion

Extant literature reveals differences in brain functional connectivity associated with a history of adverse events (for reviews, see [26, 118, 119, 135]). While many of these events are related to low SES, situations that co-occur with low SES, such as exposure to violence, are notoriously difficult to address. Treating low SES as a monolithic construct may also make it difficult to gain traction on the question of how exactly SES-related experiences impact the brain. Here we have shown that a history of exposure to violence, as distinct from low SES, is associated with reduced connectivity between regions previously shown to be important for emotion regulation and cognitive control. These results suggest that neural circuitry related to emotion regulation and cognitive control can change due to exposure to violence in particular, pointing to the possibility that future interventions could provide enhanced benefits by targeting exposure to violence, rather than having to contend with low SES in general. For example, interventions aimed at improving cognitive control may be beneficial for individuals in high-crime neighborhoods. Overall, this study demonstrates differences in neural connectivity due to exposure to violence, suggesting a neural basis for targeting the development of practical interventions.

## Supplementary Information

**Additional file 1: Figure S1.** L = left hemisphere, R = right hemisphere. Whole-brain effects of left and right amygdala seeds, for the high exposure to violence and low exposure to violence groups. Warm colors represent positive correlations, in which the amygdala seed regions are positively connected with the areas highlighted. **Figure S2.** Areas where functional connectivity strength (beta-weights) is significantly correlated with exposure to violence, after statistically controlling for current socio-economic status. A: Functional connectivity with left amygdala seed. B: Functional connectivity with right amygdala seed. Cool colors represent negative correlations, where increasing exposure to violence is associated with decreasing connectivity between each amygdala seed region and the areas highlighted. **Figure S3.** Areas where connectivity strength (beta-weights) is significantly correlated with exposure to violence, after statistically controlling for current socio-economic status. Cool colors represent negative correlations, where increasing exposure to violence is associated with decreasing connectivity between the right dlPFC seed region and the areas highlighted. **Figure S4.** Analysis of right dlPFC connectivity with seed from the Owen et al. [75] meta-analysis of working memory. Cool colors represent negative correlations, where increasing exposure to violence is associated with decreasing connectivity between the right dlPFC seed from Owen et al. [75] and the areas highlighted. Note that results are nearly identical to those in Figure 3.

## Data Availability

Restrictions apply to the datasets: The datasets for this manuscript are not publicly available because of IRB requirements for confidentiality. Requests to access the datasets should be directed to William W. Graves, william.graves@rutgers.edu.

## References

[1] Clark C, Ryan L, Kawachi I, Canner MJ, Berkman L, Wright RJ (2008). Witnessing community violence in residential neighborhoods: a mental health hazard for urban women. J Urban Health.

[2] Sharkey P, Schwartz AE, Ellen IG, Lacoe J (2014). High stakes in the classroom, high stakes on the street: the effects of community violence on student’s standardized test performance. Sociol Sci.

[3] Sharkey PT, Tirado-Strayer N, Papachristos AV, Raver CC (2012). The effect of local violence on children’s attention and impulse control. Am J Public Health.

[4] Lynch M (2003). Consequences of children’s exposure to community violence. Clin Child Fam Psychol Rev.

[5] Boxer P, Sloan-Power E (2013). Coping with violence: a comprehensive framework and implications for understanding resilience. Trauma Violence Abus.

[6] Strenziok M, Krueger F, Deshpande G, Lenroot RK, Van der meer E, Grafman J. (2011). Fronto-parietal regulation of media violence exposure in adolescents a multi-method study. Soc Cogn Affect Neurosci.

[7] Hummer TA (2015). Media violence effects on brain development. Am Behav Sci.

[8] Zvyagintsev M, Klasen M, Weber R, Sarkheil P, Esposito F, Mathiak KA (2016). Violence-related content in video game may lead to functional connectivity changes in brain networks as revealed by fMRI-ICA in young men. Neuroscience.

[9] Cará VM, Esper NB, De Azeredo LA, Iochpe V, Dalfovo NP, Santos RC (2019). An fMRI study of inhibitory control and the effects of exposure to violence in Latin-American early adolescents: alterations in frontoparietal activation and performance. Soc Cogn Affect Neurosci.

[10] Hart H, Lim L, Mehta MA, Chatzieffraimidou A, Curtis C, Xu X (2017). Reduced functional connectivity of fronto-parietal sustained attention networks in severe childhood abuse. PLoS ONE.

[11] Thomason ME, Marusak HA, Tocco MA, Vila AM, McGarragle O, Rosenberg DR (2015). Altered amygdala connectivity in urban youth exposed to trauma. Soc Cogn Affect Neurosci.

[12] Biswal B, Zerrin Yetkin F, Haughton VM, Hyde JS (1995). Functional connectivity in the motor cortex of resting human brain using echo-planar mri. Magn Reson Med.

[13] Friston KJ (1994). Functional and effective connectivity in neuroimaging: a synthesis. Hum brain mapp.

[14] van den Heuvel MP, Hulshoff Pol HE (2010). Exploring the brain network: a review on resting-state fMRI functional connectivity. Eur Neuropsychopharmacol.

[15] Goldin PR, McRae K, Ramel W, Gross JJ (2008). The neural bases of emotion regulation: reappraisal and suppression of negative emotion. Biol Psychiatry.

[16] Kohn N, Eickhoff SB, Scheller M, Laird AR, Fox PT, Habel U (2014). Neural network of cognitive emotion regulation—an ALE meta-analysis and MACM analysis. Neuroimage.

[17] McEwen BS, Gianaros PJ (2010). Central role of the brain in stress and adaptation: links to socioeconomic status, health, and disease. Ann N Y Acad Sci.

[18] Bickel WK, Moody L, Quisenberry AJ, Ramey CT, Sheffer CE (2014). A competing neurobehavioral decision systems model of SES-related health and behavioral disparities. Prev Med (Baltim).

[19] Farah MJ, Shera DM, Savage JH, Betancourt L, Giannetta JM, Brodsky NL (2006). Childhood poverty: specific associations with neurocognitive development. Brain Res.

[20] Pechtel P, Pizzagalli DA (2011). Effects of early life stress on cognitive and affective function: an integrated review of human literature. Psychopharmacology (Berl).

[21] Evans GW, Kim P (2010). Multiple risk exposure as a potential explanatory mechanism for the socioeconomic status-health gradient. Ann N Y Acad Sci.

[22] Malter Cohen M, Jing D, Yang RR, Tottenham N, Lee FS, Casey BJ (2013). Early-life stress has persistent effects on amygdala function and development in mice and humans. Proc Natl Acad Sci.

[23] Herzberg MP, Gunnar MR (2020). Early life stress and brain function: activity and connectivity associated with processing emotion and reward. NeuroImage.

[24] Tottenham N, Hare TA, Quinn BT, McCarry TW, Nurse M, Gilhooly T (2010). Prolonged institutional rearing is associated with atypically large amygdala volume and difficulties in emotion regulation. Dev Sci.

[25] Tottenham N, Hare TA, Millner A, Gilhooly T, Zevin JD, Casey BJ (2011). Elevated amygdala response to faces following early deprivation. Dev Sci.

[26] Kraaijenvanger EJ, Pollok TM, Monninger M, Kaiser A, Brandeis D, Banaschewski T (2020). Impact of early life adversities on human brain functioning: a coordinate-based meta-analysis. Neurosci Biobehav Rev.

[27] Dannlowski U, Stuhrmann A, Beutelmann V, Zwanzger P, Lenzen T, Grotegerd D (2012). Limbic scars: long-term consequences of childhood maltreatment revealed by functional and structural magnetic resonance imaging. Biol Psychiatry.

[28] Johnstone T, van Reekum CM, Urry HL, Kalin NH, Davidson RJ (2007). Failure to regulate: counterproductive recruitment of top-down prefrontal-subcortical circuitry in major depression. J Neurosci.

[29] Martin NL, Delgado MR (2007). Neural correlates of positive and negative emotion regulation. J Cogn Neurosci.

[30] Ochsner KN, Silvers JA, Buhle JT (2012). Functional imaging studies of emotion regulation: a synthetic review and evolving model of the cognitive control of emotion. Ann N Y Acad Sci.

[31] Phelps EA, Delgado MR, Nearing KI, LeDoux JE (2004). Extinction learning in humans: role of the amygdala and vmPFC. Neuron.

[32] Urry HL, van Reekum CM, Johnstone T, Kalin NH, Thurow ME, Schaefer HS (2006). Amygdala and ventromedial prefrontal cortex are inversely coupled during regulation of negative affect and predict the diurnal pattern of cortisol secretion among older adults. J Neurosci.

[33] Hanson JL, Albert D, Skinner AT, Shen SH, Dodge KA, Lansford JE (2019). Resting state coupling between the amygdala and ventromedial prefrontal cortex is related to household income in childhood and indexes future psychological vulnerability to stress. Sch Resarch Creat Work Bryn Mawr Coll.

[34] Marusak HA, Thomason ME, Peters C, Zundel C, Elrahal F, Rabinak CA (2016). You say “prefrontal cortex” and I say “anterior cingulate”: meta-analysis of spatial overlap in amygdala-to-prefrontal connectivity and internalizing symptomology. Transl Psychiatry.

[35] Tottenham N, Sheridan MA (2010). A review of adversity, the amygdala and the hippocampus: a consideration of developmental timing. Front Hum Neurosci.

[36] Arnsten AFT, Raskind MA, Taylor FB, Connor DF (2015). The effects of stress exposure on prefrontal cortex: translating basic research into successful treatments for post-traumatic stress disorder. Neurobiol Stress.

[37] Moffitt TE, Arseneault L, Danese A, Fisher H, Mill J, Pariante C (2013). Childhood exposure to violence and lifelong health: clinical intervention science and stress biology research join forces. Dev Psychopathol.

[38] Boxer P, Schappell A, Middlemass K, Mercado I (2011). Cognitive and emotional covariates of violence exposure among former prisoners: links to antisocial behavior and emotional distress and implications for theory. Aggress Behav.

[39] Kliewer W, Cunningham JN, Diehl R, Parrish KA, Jean M, Atiyeh C (2004). Violence exposure and adjustment in inner-city youth: child and caregiver emotion regulation skill, caregiver—child relationship quality, and neighborhood cohesion as protective factor. J Clin Child Adolesc Psychol.

[40] Marusak HA, Martin KR, Etkin A, Thomason ME (2015). Childhood trauma exposure disrupts the automatic regulation of emotional processing. Neuropsychopharmacol.

[41] Hein TC, Monk CS (2017). Research review: neural response to threat in children, adolescents, and adults after child maltreatment—a quantitative meta-analysis. J Child Psychol Psychiatry.

[42] McCrory EJ, De Brito SA, Sebastian CL, Mechelli A, Bird G, Kelly PA (2011). Heightened neural reactivity to threat in child victims of family violence. Curr Biol.

[43] Weissman DG, Jenness JL, Colich NL, Miller AB, Sambrook KA, Sheridan MA (2020). Altered neural processing of threat-related information in children and adolescents exposed to violence: a transdiagnostic mechanism contributing to the emergence of psychopathology. J Am Acad Child Adolesc Psychiatry.

[44] Lee TW, Xue SW (2018). Does emotion regulation engage the same neural circuit as working memory? a meta-analytical comparison between cognitive reappraisal of negative emotion and 2-back working memory task. PLoS ONE.

[45] Marshall NA, Marusak HA, Sala-Hamrick KJ, Crespo LM, Rabinak CA, Thomason ME (2008). Socioeconomic disadvantage and altered corticostriatal circuitry in urban youth. Hum Brain Mapp.

[46] Carter CS, Van Veen V, Botvinick MM, Cohen JD, Stenger VA (2001). Anterior cingulate cortex, conflict monitoring, and levels of processing. Neuroimage.

[47] Bush G, Luu P, Posner MI (2000). Cognitive and emotional influences in anterior cingulate cortex. Trends Cogn Sci.

[48] Etkin A, Egner T, Peraza DM, Kandel ER, Hirsch J (2006). Resolving emotional conflict: a role for the rostral anterior cingulate cortex in modulating activity in the amygdala. Neuron.

[49] Hummer TA, Kronenberger WG, Wang Y, Mathews VP. Decreased prefrontal activity during a cognitive inhibition task following violent video game play: A multi-week randomized trial. Psychology of Popular Media Culture. 2019;8(1).

[50] Bos KJ, Fox N, Zeanah CH, Nelson CA (2009). Effects of early psychosocial deprivation on the development of memory and executive function. Front Behav Neurosci.

[51] De Bellis MD, Hooper SR, Spratt EG, Woolley DP (2009). Neuropsychological findings in childhood neglect and their relationships to pediatric PTSD. J Int Neuropsychol Soc.

[52] Goodman JB, Freeman EE, Chalmers KA (2019). The relationship between early life stress and working memory in adulthood: a systematic review and meta-analysis. Memory.

[53] Thomas Yeo BT, Krienen FM, Sepulcre J, Sabuncu MR, Lashkari D, Hollinshead M (2011). The organization of the human cerebral cortex estimated by intrinsic functional connectivity. J Neurophysiol.

[54] Power JD, Cohen AL, Nelson SM, Wig GS, Barnes KA, Church JA (2011). Functional network organization of the human brain. Neuron.

[55] Sheridan MA, Peverill M, Finn AS, McLaughlin KA (2017). Dimensions of childhood adversity have distinct associations with neural systems underlying executive functioning. Dev Psychopathol.

[56] Qin SZ, Hermans EJ, van Marle HJFF, Luo J, Fernández G, Fernandez G (2009). Acute psychological stress reduces working memory-related activity in the dorsolateral prefrontal cortex. Biol Psychiatry.

[57] Sheridan MA, McLaughlin KA (2014). Dimensions of early experience and neural development: deprivation and threat. Trends Cogn Sci.

[58] Sheridan MA, Shi F, Miller AB, Salhi C, McLaughlin KA (2020). Network structure reveals clusters of associations between childhood adversities and development outcomes. Dev Sci.

[59] Schmeichel BJ, Demaree HA (2010). Working memory capacity and spontaneous emotion regulation: high capacity predicts self-enhancement in response to negative feedback. Emotion.

[60] Coifman K, Matt LM, Nylocks KM, Aurora P (2019). Predicting negative affect variability and spontaneous emotion regulation: can working memory span tasks estimate emotion regulatory capacity?. Emotion.

[61] Barkus E (2020). Effects of working memory training on emotion regulation: Transdiagnostic review. PsyCh J.

[62] Xiu L, Zhou R, Jiang Y (2016). Working memory training improves emotion regulation ability: evidence from HRV. Physiol Behav.

[63] Richters J, Saltzman W. Screening Survey of Exposure to Community Violence: Self-Report Version. Rockville, MD: National Institute of Mental Health. 1990.

[64] Team RC. Software for data analysis: programming with {R}. R foundation for statistical computing. Vienna, Austria: Springer-Verlag. 2020. (Statistics and Computing).

[65] Richters JE, Martinez P (1993). The NIMH community violence project: I. children as victims of and witnesses to violence. Psychiatry.

[66] Kuppuswamy B (1981). Manual of socioeconomic status scale (urban). Delhi Manasayan.

[67] Gallo LC, Bogart LM, Vranceanu A-M, Matthews KA (2005). Socioeconomic status, resources, psychological experiences, and emotional responses: a test of the reserve capacity model. J Pers Soc Psychol.

[68] Lancaster JL, Woldorff MG, Parsons LM, Liotti M, Freitas CS, Rainey L (2000). Automated talairach atlas labels for functional brain mapping. Hum Brain Mapp.

[69] Philip NS, Valentine TR, Sweet LH, Tyrka AR, Price LH, Carpenter LL (2014). Early life stress impacts dorsolateral prefrontal cortex functional connectivity in healthy adults: informing future studies of antidepressant treatments. J Psychiatr Res.

[70] Hummer TA, Wang Y, Kronenberger WG, Mosier KM, Kalnin AJ, Dunn DW (2010). Short-term violent video game play by adolescents alters prefrontal activity during cognitive inhibition. Media Psychol.

[71] Paquola C, Bennett MR, Lagopoulos J (2016). Understanding heterogeneity in grey matter research of adults with childhood maltreatment—a meta-analysis and review. Neurosci Biobehavioral Rev.

[72] Zanolie K, Van Leijenhorst L, Rombouts SARB, Crone EA (2008). Separable neural mechanisms contribute to feedback processing in a rule-learning task. Neuropsychologia.

[73] Cox RW (1996). AFNI: software for analysis and visualization of functional magnetic resonance neuroimages. Comput Biomed Res.

[74] Lacadie CM, Fulbright RK, Constable RT, Papademetris X (2008). More accurate talairach coordinates for neuroimaging using nonlinear registration. Neuroimage.

[75] Owen AM, McMillan KM, Laird AR, Bullmore E (2005). N-back working memory paradigm: a meta-analysis of normative functional neuroimaging studies. Hum Brain Mapp.

[76] Nee DE, Brown JW, Askren MK, Berman MG, Demiralp E, Krawitz A (2013). A meta-analysis of executive components of working memory. Cereb Cortex.

[77] Rottschy C, Langner R, Dogan I, Reetz K, Laird AR, Schulz JB (2012). Modelling neural correlates of working memory: a coordinate-based meta-analysis. Neuroimage.

[78] Saad ZS, Glen DR, Chen G, Beauchamp MS, Desai R, Cox RW (2009). A new method for improving functional-to-structural MRI alignment using local pearson correlation. Neuroimage.

[79] Talairach J, Tournoux P (1988). Co-planar stereotaxic axis of the human brain.

[80] Nichols T, Brett M, Andersson J, Wager T, Poline JB (2005). Valid conjunction inference with the minimum statistic. Neuroimage.

[81] Lupien SJ, McEwen BS, Gunnar MR, Heim C (2009). Effects of stress throughout the lifespan on the brain, behaviour and cognition. Nat Rev Neurosci.

[82] McLaughlin KA, Weissman D, Bitrán D (2019). Childhood adversity and neural development: a systematic review. Annu Rev Dev Psychol.

[83] Fan Y, Pestke K, Feeser M, Aust S, Pruessner JC, Böker H (2015). Amygdala-hippocampal connectivity changes during acute psychosocial stress: joint effect of early life stress and oxytocin. Neuropsychopharmacol.

[84] Gee DG, Humphreys KL, Flannery J, Goff B, Telzer EH, Shapiro M (2013). A developmental shift from positive to negative connectivity in human amygdala-prefrontal circuitry. J Neurosci.

[85] Nooner KB, Mennes M, Brown S, Castellanos FX, Leventhal B, Milham MP (2013). Relationship of trauma symptoms to amygdala-based functional brain changes in adolescents. J Trauma Stress.

[86] Kumar P, Berghorst LH, Nickerson LD, Dutra SJ, Goer FK, Greve DN (2014). Differential effects of acute stress on anticipatory and consummatory phases of reward processing. Neuroscience.

[87] Oei NYL, Veer IM, Wolf OT, Spinhoven P, Rombouts SRB, Elzinga BM (2012). Stress shifts brain activation towards ventral “affective” areas during emotional distraction. Soc Cogn Affect Neurosci.

[88] Banks SJ, Eddy KT, Angstadt M, Nathan PJ, Phan KL (2007). Amygdala–frontal connectivity during emotion regulation. Soc Cogn Affect Neurosci.

[89] Kim P, Evans GW, Angstadt M, Ho SS, Sripada CS, Swain JE (2013). Effects of childhood poverty and chronic stress on emotion regulatory brain function in adulthood. Proc Natl Acad Sci.

[90] Treadway MT, Buckholtz JW, Zald DH (2013). Perceived stress predicts altered reward and loss feedback processing in medial prefrontal cortex. Front Hum Neurosci.

[91] Hare TA, Tottenham N, Galvan A, Voss HU, Glover GH, Casey BJ (2008). Biological substrates of emotional reactivity and regulation in adolescence during an emotional go-nogo task. Biol Psychiatry.

[92] Shin LM, Wright CI, Cannistraro PA, Wedig MM, McMullin K, Martis B (2005). A functional magnetic resonance imaging study of amygdala and medial prefrontal cortex responses to overtly presented fearful faces in posttraumatic stress disorder. Arch Gen Psychiatry.

[93] Eluvathingal TJ, Chugani HT, Behen ME, Juhász C, Muzik O, Maqbool M (2006). Abnormal brain connectivity in children after early severe socioemotional deprivation: a diffusion tensor imaging study. Pediatrics.

[94] Kier EL, Staib LH, Davis LM, Bronen RA (2004). MR imaging of the temporal stem: anatomic dissection tractography of the uncinate fasciculus, inferior occipitofrontal fasciculus, and Meyer’s Loop of the optic radiation. AJNR Am J Neuroradiol.

[95] Tsavoussis A, Stawicki SPA, Stoicea N, Papadimos TJ (2014). Child-witnessed domestic violence and its adverse effects on brain development: a call for societal self-examination and awareness. Front public Heal.

[96] Manoach DS, Schlaug G, Siewert B, Darby DG, Bly BM, Benfield A (1997). Prefrontal cortex fMRI signal changes are correlated with working memory load. NeuroReport.

[97] Cole MW, Reynolds JR, Power JD, Repovs G, Anticevic A, Braver TS (2013). Multi-task connectivity reveals flexible hubs for adaptive task control. Nat Neurosci.

[98] Mizoguchi K, Yuzurihara M, Ishige a, Sasaki H, Chui DH, Tabira T (2000). Chronic stress induces impairment of spatial working memory because of prefrontal dopaminergic dysfunction. J Neurosci.

[99] Philip NS, Sweet LH, Tyrka AR, Carpenter SL, Albright SE, Price LH (2016). Exposure to childhood trauma is associated with altered n-back activation and performance in healthy adults: implications for a commonly used working memory task. Brain Imaging Behav.

[100] McLaughlin KA, Sheridan MA, Gold AL, Duys A, Lambert HK, Peverill M (2016). Maltreatment exposure, brain structure, and fear conditioning in children and adolescents. Neuropsychopharmacology.

[101] Cassiers LLM, Sabbe BGC, Schmaal L, Veltman DJ, Penninx BWJH, Van Den Eede F (2018). Structural and functional brain abnormalities associated with exposure to different childhood trauma subtypes: a systematic review of neuroimaging findings. Front Psychiatry.

[102] Herringa RJ, Birn RM, Ruttle PL, Burghy CA, Stodola DE, Davidson RJ (2013). Childhood maltreatment is associated with altered fear circuitry and increased internalizing symptoms by late adolescence. Proc Natl Acad Sci.

[103] Van Essen DC (1997). A tension-based theory of morphogenesis and compact wiring in the central nervous system. Nature.

[104] Hua JPY, Trull TJ, Merrill AM, McCarty RM, Straub KT, Kerns JG (2020). Daily-life affective instability in emotional distress disorders is associated with function and structure of posterior parietal cortex. Psychiatry Res Neuroimag.

[105] McLaughlin KA, Lambert HK (2017). Child trauma exposure and psychopathology: mechanisms of risk and resilience. Curr Opin Psychol.

[106] Hartley CA, Phelps EA (2010). Changing fear: the neurocircuitry of emotion regulation. Neuropsychopharmacology.

[107] Fan J, Flombaum JI, McCandliss BD, Thomas KM, Posner MI (2003). Cognitive and brain consequences of conflict. Neuroimage.

[108] Liu X, Banich MT, Jacobson BL, Tanabe JL (2004). Common and distinct neural substrates of attentional control in an integrated Simon and spatial Stroop task as assessed by event-related fMRI. Neuroimage.

[109] Ridderinkhof KR, van den Wildenberg WPM, Segalowitz SJ, Carter CS (2004). Neurocognitive mechanisms of cognitive control: the role of prefrontal cortex in action selection, response inhibition, performance monitoring, and reward-based learning. Brain Cogn.

[110] Quirk GJ, Paré D, Richardson R, Herry C, Monfils MH, Schiller D (2010). Erasing fear memories with extinction training. J Neurosci.

[111] Etkin A, Egner T, Kalisch R (2011). Emotional processing in anterior cingulate and medial prefrontal cortex. Trends Cogn Sci.

[112] Vogt BA (2016). Midcingulate cortex: structure, connections, homologies, functions and diseases. J Chem Neuroanat.

[113] Ochsner KN, Gross JJ (2005). The cognitive control of emotion. Trends Cogn Sci.

[114] Pessoa L (2009). How do emotion and motivation direct executive control?. Trends Cogn Sci.

[115] Milham M, Banich M, Webb A, Barad V, Cohen N, Wszalek T (2001). The relative involvement of anterior cingulate and prefrontal cortex in attentional control depends on nature of conflict. Cogn Brain Res.

[116] Carter CS, Braver TS, Barch DM, Botvinick MM, Noll D, Cohen JD (1998). Anterior cingulate cortex, error detection, and the online monitoring of performance. Science.

[117] Kujawa A, Wu M, Klumpp H, Pine DS, Swain JE, Fitzgerald KD (2016). Altered development of amygdala-anterior cingulate cortex connectivity in anxious youth and young adults. Biol Psychiatry Cogn Neurosci Neuroimag.

[118] Dark HE, Harnett NG, Goodman AM, Wheelock MD, Mrug S, Schuster MA (2020). Violence exposure, affective style, and stress-induced changes in resting state functional connectivity. Cogn Affect Behav Neurosci.

[119] Dedovic K, D’Aguiar C, Pruessner JC (2009). What stress does to your brain: a review of neuroimaging studies. Can J Psychiatry.

[120] Spinelli S, Chefer S, Suomi SJ, Higley JD, Barr CS, Stein E (2009). Early-life stress induces long-term morphologic changes in primate brain. Arch Gen Psychiatry.

[121] Rubin M, Denson N, Kilpatrick S, Matthews KE, Stehlik T, Zyngier D (2014). “I am working-class”: subjective self-definition as a missing measure of social class and socioeconomic status in higher education research. Educ Res.

[122] Philip NS, Sweet LH, Tyrka AR, Price LH, Bloom RF, Carpenter LL (2013). Decreased default network connectivity is associated with early life stress in medication-free healthy adults. Eur Neuropsychopharmacol.

[123] Hauser MD (2021). How early life adversity transforms the learning brain. Mind Brain Educ.

[124] Walker SP, Wachs TD, Grantham-McGregor S, Black MM, Nelson CA, Huffman SL (2011). Inequality in early childhood: risk and protective factors for early child development. Lancet.

[125] Oomen CA, Soeters H, Audureau N, Vermunt L, Van Hasselt FN, Manders EMM (2010). Behavioral/systems/cognitive severe early life stress hampers spatial learning and neurogenesis, but improves hippocampal synaptic plasticity and emotional learning under high-stress conditions in adulthood. J Neurosci.

[126] Morgenroth E, Saviola F, Gilleen J, Allen B, Lührs M, Eysenck WM (2020). Using connectivity-based real-time fMRI neurofeedback to modulate attentional and resting state networks in people with high trait anxiety. NeuroImage Clin.

[127] Sampson RJ (1997). Neighborhoods and violent crime: a multilevel study of collective efficacy. Science.

[128] Krieger N, Rowley DL, Herman AA, Avery B (1993). Racism, sexism, and social class: implications for studies of health, disease, and well-being. Am J Prev Med.

[129] Molnar BE, Gortmaker SL, Bull FC, Buka SL (2004). Unsafe to play? neighborhood disorder and lack of safety predict reduced physical activity among urban children and adolescents. Am J Heal Promot.

[130] Sinha R (2008). Chronic stress, drug use, and vulnerability to addiction. Ann N Y Acad Sci.

[131] Mackey AP, Miller Singley AT, Wendelken C, Bunge SA (2015). Characterizing behavioral and brain changes associated with practicing reasoning skills. PLoS ONE.

[132] Myers CA, Wang C, Black JM, Bugescu N, Hoeft F (2016). The matter of motivation: striatal resting-state connectivity is dissociable between grit and growth mindset. Soc Cogn Affect Neurosci.

[133] van Marle HJF, Hermans EJ, Qin S, Fernández G (2009). From specificity to sensitivity: how acute stress affects amygdala processing of biologically salient stimuli. Biol Psychiatry.

[134] Hanson JL, Williams AV, Bangasser DA, Peña CJ (2021). Impact of early life stress on reward circuit function and regulation. Front Psychiatry.

[135] McLaughlin K. 2020 Early Life Stress and Psychopathology. In: The Oxford Handbook of Stress and Mental Health. 10.1093/oxfordhb/9780190681777.001.0001.

