# Supplementary material for: Exposure to violence is associated with decreased neural connectivity in emotion regulation and cognitive control, but not working memory, networks after accounting for socioeconomic status: a preliminary study
Source: Behav Brain Funct. 2022 Dec 12;18:15. doi: 10.1186/s12993-022-00201-8 (PMC9743673; doi:10.1186/s12993-022-00201-8)
Supplement: Supplementary file 1 — Additional file 1: Figure S1. L = left hemisphere, R = right hemisphere. Whole-brain effects of left and right amygdala seeds, for the high exposure to violence and low exposure to violence groups. Warm colors represent positive correlations, in which the amygdala seed regions are positively connected with the areas highlighted. Figure S2. Areas where functional connectivity strength (beta-weights) is significantly correlated with exposure to violence, after statistically controlling for current socio-economic status. A: Functional connectivity with left amygdala seed. B: Functional connectivity with right amygdala seed. Cool colors represent negative correlations, where increasing exposure to violence is associated with decreasing connectivity between each amygdala seed region and the areas highlighted. Figure S3. Areas where connectivity strength (beta-weights) is significantly correlated with exposure to violence, after statistically controlling for current socio-economic status. Cool colors represent negative correlations, where increasing exposure to violence is associated with decreasing connectivity between the right dlPFC seed region and the areas highlighted. Figure S4. Analysis of right dlPFC connectivity with seed from the Owen et al. [75] meta-analysis of working memory. Cool colors represent negative correlations, where increasing exposure to violence is associated with decreasing connectivity between the right dlPFC seed from Owen et al. [75] and the areas highlighted. Note that results are nearly identical to those in Figure 3. [file 12993_2022_201_MOESM1_ESM.docx]

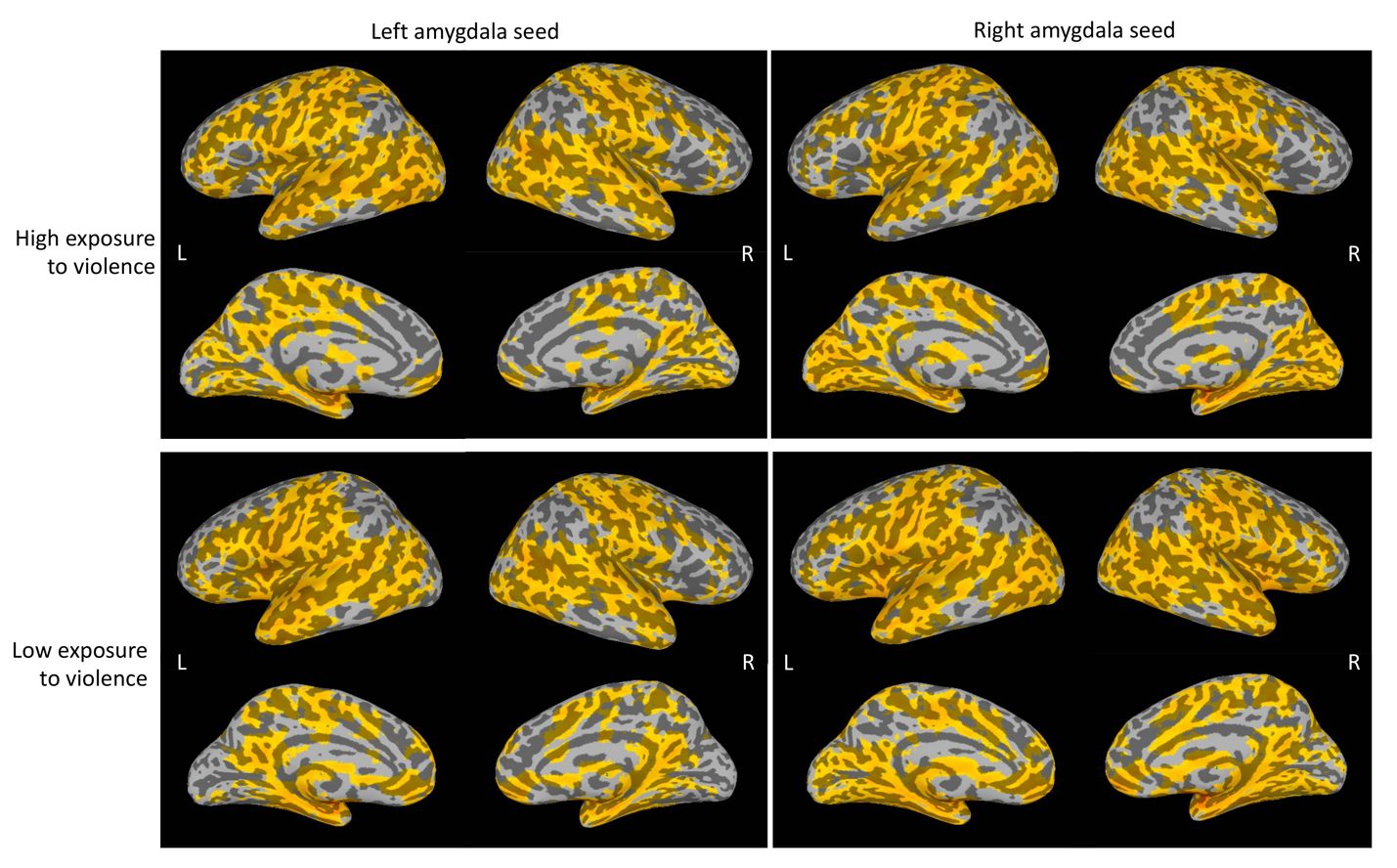


Additional file: Figure 1: L = left hemisphere, R = right hemisphere. Whole-brain effects of left and right amygdala seeds, for the high exposure to violence and low exposure to violence groups. Warm colors represent positive correlations, in which the amygdala seed regions are positively connected with the areas highlighted.


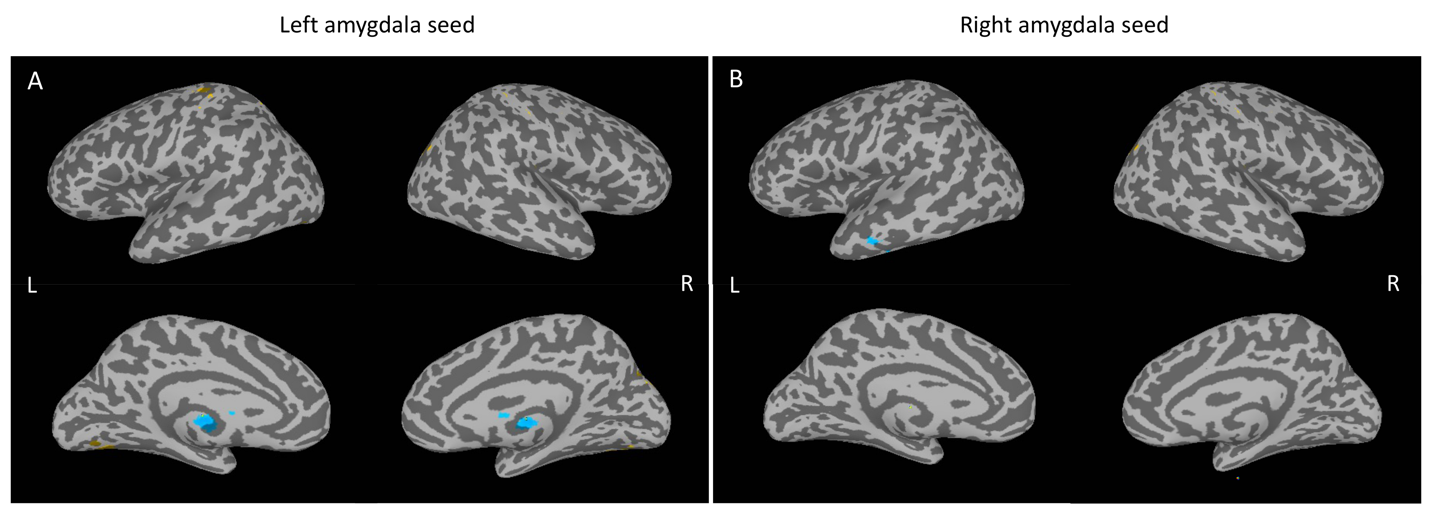


Additional file: Figure 2: Areas where functional connectivity strength (beta-weights) is significantly correlated with exposure to violence, after statistically controlling for current socio-economic status. A: Functional connectivity with left amygdala seed. B: Functional connectivity with right amygdala seed. Cool colors represent negative correlations, where increasing exposure to violence is associated with decreasing connectivity between each amygdala seed region and the areas highlighted.


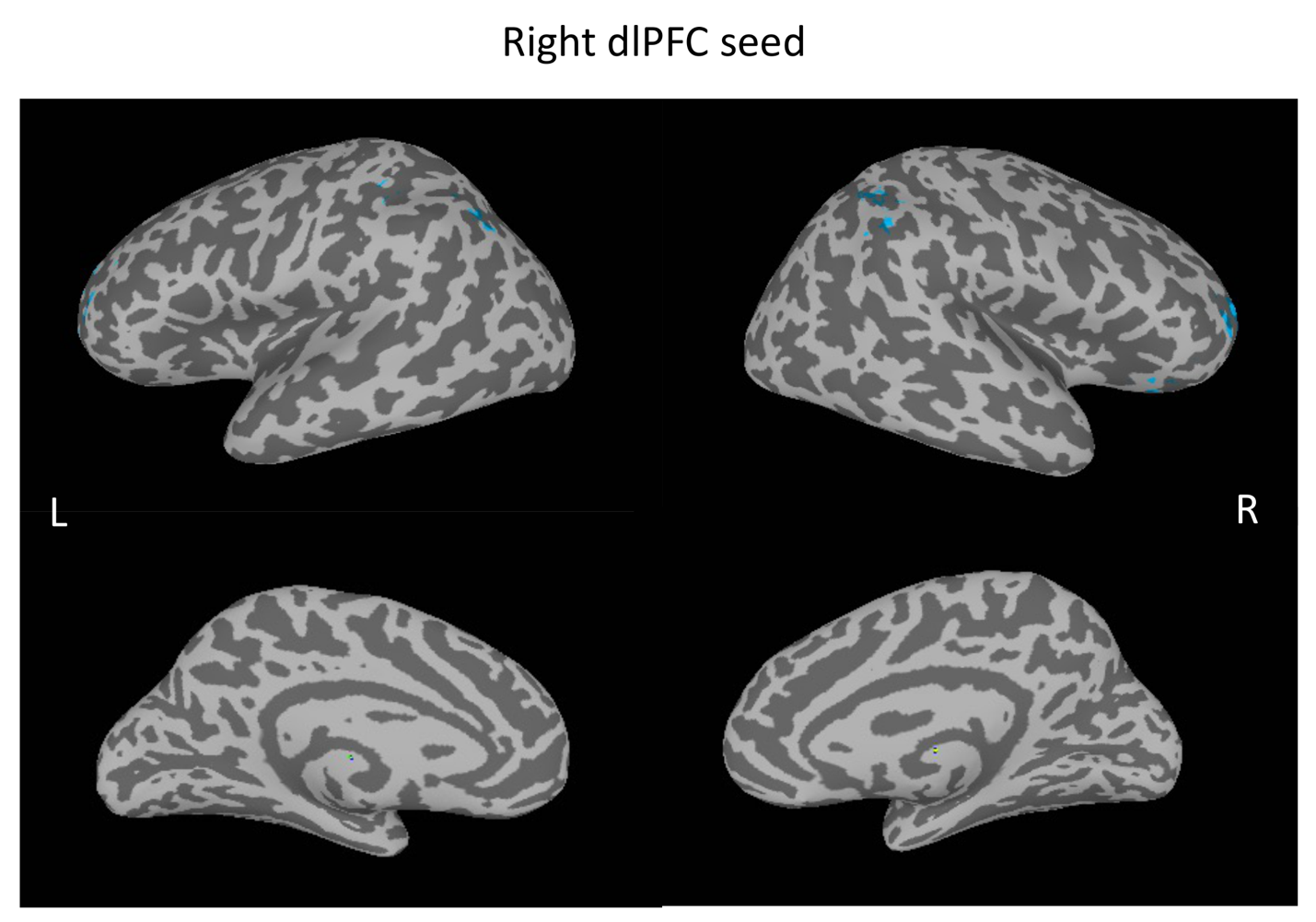


Additional file: Figure 3: Areas where connectivity strength (beta-weights) is significantly correlated with exposure to violence, after statistically controlling for current socio-economic status. Cool colors represent negative correlations, where increasing exposure to violence is associated with decreasing connectivity between the right dlPFC seed region and the areas highlighted.


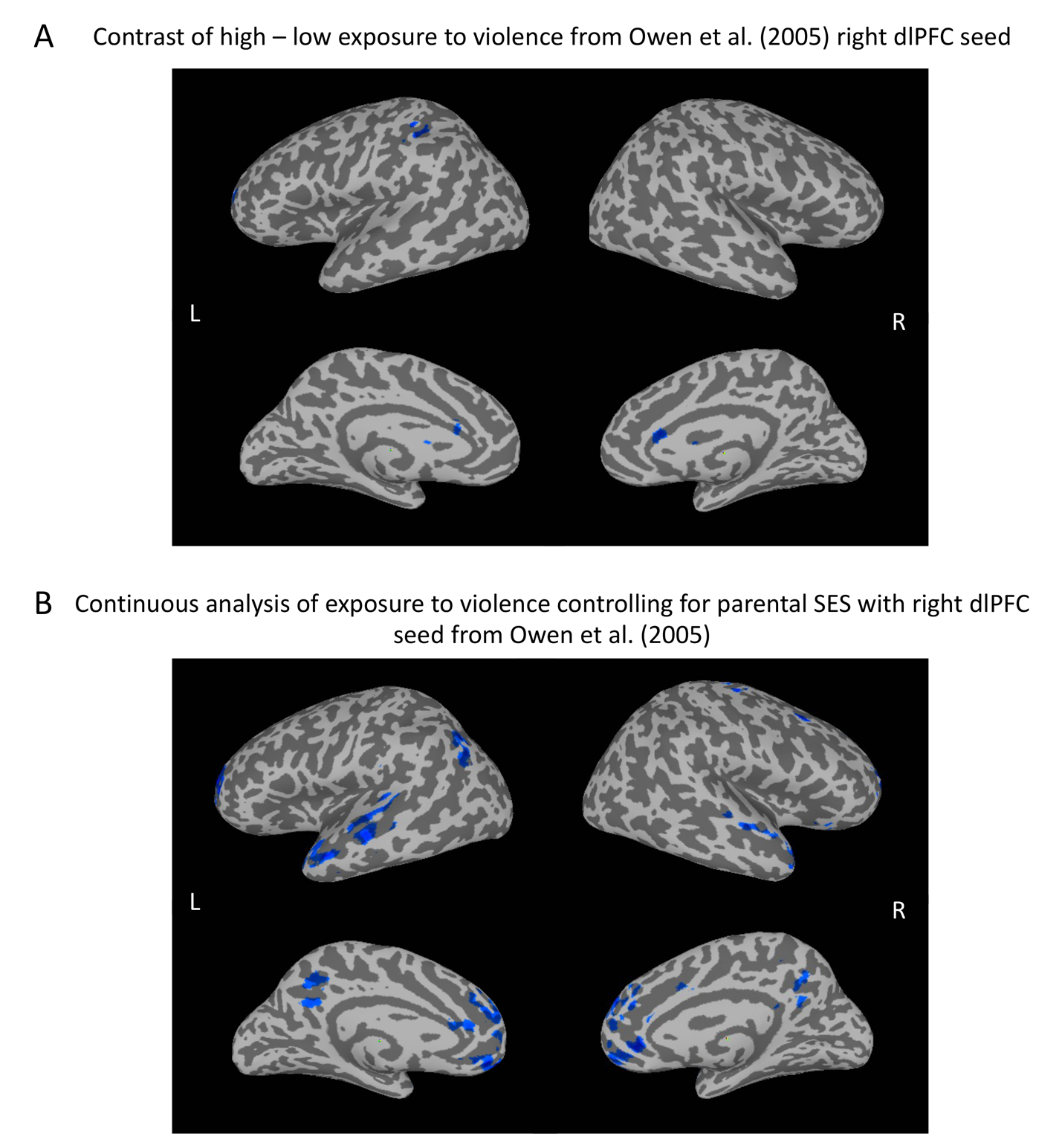


Additional file: Figure 4: Analysis of right dlPFC connectivity with seed from the Owen et al. (2005) meta-analysis of working memory. Cool colors represent negative correlations, where increasing exposure to violence is associated with decreasing connectivity between the right dlPFC seed from Owen et al. (2005) and the areas highlighted. Note that results are nearly identical to those in Figure 3.
